# Universal amplification and sequencing of foot-and-mouth disease virus complete genomes using nanopore technology

**DOI:** 10.1186/s12864-025-11938-7

**Published:** 2025-08-22

**Authors:** Andrew E. Shaw, Kebaneilwe Lebani, Lina González Gordon, Ugonna E. Ihearahu, Jemma Wadsworth, Hayley M. Hicks, Noemi Polo, Graham Freimanis, Dennis Muhanguzi, Chandana Tennakoon, Richard J. Orton, Nick J. Knowles, Antonello Di Nardo, Ryan A. Waters, Barend MdeC Bronsvoort, Donald P. King

**Affiliations:** 1 The Pirbright Institute, Ash Road, Pirbright, Surrey GU24 0NF UK; 2 School of Life Sciences, Botswana International University of Science and Technology, Palapye, Botswana; 3 Division of Epidemiology, Roslin Institute and Royal (Dick) School of Veterinary Studies, The University of Edinburgh, Easter Bush Campus, Midlothian, EH25 9RG UK; 4 Makerere University, 7062 University Rd, Kampala, Uganda; 5 MRC-University of Glasgow Centre for Virus Research, University of Glasgow, Garscube Campus, Glasgow, G61 1QH United Kingdom

**Keywords:** Foot-and-mouth disease, Nanopore, Sequencing, MinION, Whole genome sequencing, Complete genome

## Abstract

**Background:**

Foot-and-mouth disease virus (FMDV) is capable of causing explosive outbreaks among domestic and wild cloven-hoofed animals. Genomic characterisation of FMDV is a crucial component of disease control enabling accurate tracing of disease outbreaks to be undertaken. Nanopore sequencing is an affordable and accessible form of high-throughput sequencing (HTS) technology. However, most published methods for FMDV only sequence genomic fragments or focus upon specific lineages. In this study, a universal FMDV sequencing protocol was developed alongside a bespoke analytical pipeline to sequence any FMDV genome in the absence of prior knowledge regarding the identity of the serotype or lineage.

**Methods:**

Universal multiplex RT-PCRs were used to amplify overlapping tiles encompassing the entire FMDV genome. The PCR products were pooled and subjected to nanopore sequencing using the portable MinION sequencing device. A bioinformatics pipeline was used to assemble genomes based upon blastn and reference assembly.

**Results:**

Iterative changes in primer design and pooling resulted in two panels of primers; one set amplifying twenty short fragments (S_scheme), and another set amplifying six longer fragments (L_scheme). Both approaches were shown to be capable of generating FMDV genomes, however the L_scheme was simpler, more reliable and more cost-effective at generating complete genomes. The final L_scheme protocol was assessed using 30 FMDV isolates representing all the currently circulating lineages of FMDV. As part of the development, we successfully trialled the use of this technology in Uganda, a country endemic for FMD.

**Conclusions:**

The amplification, sequencing and bioinformatics strategy developed here has been assessed using a diverse array of FMDV lineages. Using two multiplex PCR reactions, this approach can successfully generate complete genomes of FMDV in a lineage agnostic fashion. Therefore, the primer sets and approaches described here represent a useful tool for expanding the capacity of laboratories to characterise FMDV at the genomic level.

**Supplementary Information:**

The online version contains supplementary material available at 10.1186/s12864-025-11938-7.

## Background

Foot-and-mouth disease (FMD) is one of the most economically consequential viral diseases of livestock [1]. The disease is caused by FMD virus (FMDV), species *Aphthovirus vesiculae*, within the family *Picornaviridae*. FMDV is highly contagious causing vesicular lesions in the mouth and on the feet of wild and domesticated cloven-hoofed animals including cattle, sheep, goats, and pigs. Whilst mortality is usually low among adult animals, FMDV infection can result in severe production losses.

FMDV exists as seven antigenically distinct serotypes. Serotypes O, A, Asia 1 and Southern African Territories (SAT) 1, 2 and 3 all circulate in endemic regions. In contrast, serotype C has not been detected anywhere since 2004 and is considered to be extinct [2]. The epidemiology of FMDV is complex and is underpinned by endemic circulation of different virus serotypes in seven global ‘pools’ (pool 1 – pool 7), each of which maintains a unique constellation of circulating lineages [3]. Unpredictable inter-pool movements of FMDVs further complicate the epidemiological situation [4–7] since the rapid spread of novel serotypes and lineages within a region can be facilitated by the lack of existing immunity in susceptible hosts derived from previous infection and/or vaccination. Indeed, the dynamic nature of FMD epidemiology has recently been exemplified by outbreaks in formerly FMD-free regions, for example recent outbreaks in Europe and Indonesia [8]. These factors reinforce the importance of work undertaken to continually characterise FMD viruses collected from field cases of FMD to monitor the regional circulation of FMDV lineages and the emergence of new antigenic variants.

Genetically, most serotypes encompass multiple ‘topotypes’, which comprise viral lineages (and sub-lineages) that are specific to the global regions in which they are found [2]. The genetic diversity of FMDV is further heightened by the high error rate associated with the viral RNA dependent RNA polymerase [6]. Consequently, generating molecular assays to characterise FMDV at the lineage, serotype or species level can be extremely challenging.

Differential lineage detection and characterisation can be achieved using lineage-specific real-time RT-PCR assays that have been developed for a broad range of FMDV lineages [9–14]. However, these tests do not provide data that can be used to define the evolutionary origin of FMDVs or the precise genotype of viruses. The VP1-encoding 1D region of the FMDV genome is favoured for phylogenetic trees as its nucleotide variability and lack of recombination allow the robust reconstruction of evolutionary relationships. The generation of complete genomes as opposed to routine VP1-coding sequences offers multiple advantages, for example the identification of features within non-structural proteins which may relate to other facets of viral fitness, pathogenicity, host specificity [15] or transmission [16]. Furthermore, the sequencing of complete RNA virus genomes can provide important data for disease control to allow transmission pathways to be resolved, with fine scale outbreak tracing applied to FMDV as well as multiple other viruses, including Ebolavirus, yellow fever virus, Zika virus, influenza virus and SARS-CoV-2 [17–26].

Historically, Sanger as well as short-read Illumina-based sequencing have been the dominant platforms used to sequence FMDV genomes [27, 28]. However, Illumina and Sanger sequencing technologies both suffer from the requirement for expensive hardware and dedicated infrastructure. Nanopore-based sequencing has been demonstrated as a suitable method which can be used to partially characterise FMDV [29–32]. Although direct sequencing of viral RNA has been reported (including for FMDV) [33–35], the majority of protocols that use nanopore chemistry to sequence viruses amplify fragments of the genome using PCR as a form of enrichment above the background nucleic acid, e.g. host DNA [36]. In contrast to approaches amplifying targeted regions of the genome, many protocols use multiplex PCR to amplify overlapping tiles encompassing the genome [35, 37, 38]. Increasingly, nanopore technology is being used as the HTS method of choice to sequence amplified PCR products due to being economical, quick, and its ease of deployment [38, 39]. Here, we report the development of a universal approach to amplify and sequence complete FMDV genomes using nanopore technology.

## Results

### Primer evaluation

The lack of *a priori* knowledge regarding the serotype/topotype of a FMDV positive sample means that an approach able to accommodate the wide range of phylogenetically distinct topotypes/lineages that could be present is required. We therefore designed PCR primer sets capable of amplifying highly diverse FMD genomes. Experiments to iteratively design the primer schemes were performed using a selection of virus isolates archived at the FAO World Reference Laboratory for FMD (WRLFMD), Pirbright, Table 1.

**Table 1:** FMDV isolates used to evaluate primer combinations for the amplification of complete genomes

Isolate	Serotype	Topotype	Lineage	Pool^1^	Panel
VIT/1/2017	O	SEA	Mya-98	1	SEA
VIT/1/2018	O	ME-SA	PanAsia	1	SEA
VIT/11/2017	A	ASIA	Sea-97	1	SEA
KEN/4/2018	O	EA-2		4	EA
ETH/4/2015	O	EA-3		4	EA
ETH/9/2019	O	EA-3		4	EA
ETH/30/2016	O	EA-4		4	EA
ETH/14/2019	O	EA-4		4	EA
ETH/2/2018	A	AFRICA	G-I	4	EA
UGA/28/2019	A	AFRICA	G-I	4	EA
SUD/9/2018	A	AFRICA	G-IV	4	EA
ETH/19/2019	A	AFRICA	G-IV	4	EA
TAN/27/2012	SAT1	I		4	EA
TAN/22/2013	SAT1	I		4	EA
KEN/10/2013	SAT1	I		4	EA
TAN/22/2014	SAT1	I		4	EA
KEN/19/2017	SAT2	IV		4	EA
ETH/16/2015	SAT2	VII	Alx-12	4	EA
EGY/1/2018	SAT2	VII	Ghb-12	4	EA
ETH/11/2018	SAT2	VII	Lib-12	4	EA

*SEA* refers to virus isolates from Southeast Asia, *EA* refers to virus isolates from East Africa

^1‘^Pool’ refers to the epidemiological pools where the lineage is observed [3]

**Fig. 1:**
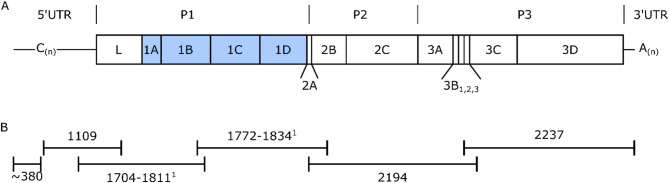
The FMDV genome. (**A**) A scaled representation of the FMDV region. The polyprotein is divided into P1, P2 and P3 and flanked by 5’ and 3’ untranslated regions (UTRs). The capsid coding regions 1A-1D are shaded blue. A poly cytosine (poly(C), (C_n_)) region separates the S fragment from the L fragment, and the 3’UTR is followed by a poly(A) tail (A_n_). (**B**) The six amplicons in the finalised L_scheme. One amplicon ~ 380 bp represents the S fragment, with five others covering the L fragment. ^1^Amplicon length depends upon the serotype being amplified

We first focused upon evaluating a primer scheme comprising 20 overlapping tiles (on average 572 bp in length) – termed the S_scheme - using the East African epidemiological pool as it contains a diverse array of serotypes and lineages, including serotypes O (4 lineages recorded), A (3 lineages recorded), SAT1 (4 lineages recorded) and SAT2 (5 lineages recorded) and thus represented a challenging scenario against which to evaluate the primers. We further evaluated the performance of the S_scheme by testing representative FMDV isolates from East and Southeast Asia. Recent isolates of viruses representing three key topotypes currently circulating within Southeast Asia (O/SEA/Mya-98, O/ME-SA/PanAsia and A/ASIA/Sea-97) were selected to assess the suitability of the primer scheme for Pool 1 viruses (Table 1). As expected, the majority of amplification failures when using initial primer designs were associated with primers that targeted the highly variable P1 region (Fig. 1A, Additional file 1 [Table S1]). Failed PCR reactions within the capsid coding region were particularly evident for serotype SAT1 where 13/24 PCRs failed across 4 viruses (Additional file 1 Table S1). Similar results were obtained for the Southeast Asian isolates, where the amplicon 6 PCR failed for A/VIT/11/2017 (Additional file 2 Table S2). In contrast, PCRs using primers located in P2 and P3 robustly amplified the target fragments. Iterative re-designs of faulty primer pairs resulted in universal amplification across the test panel of isolates.

**Fig. 2:**
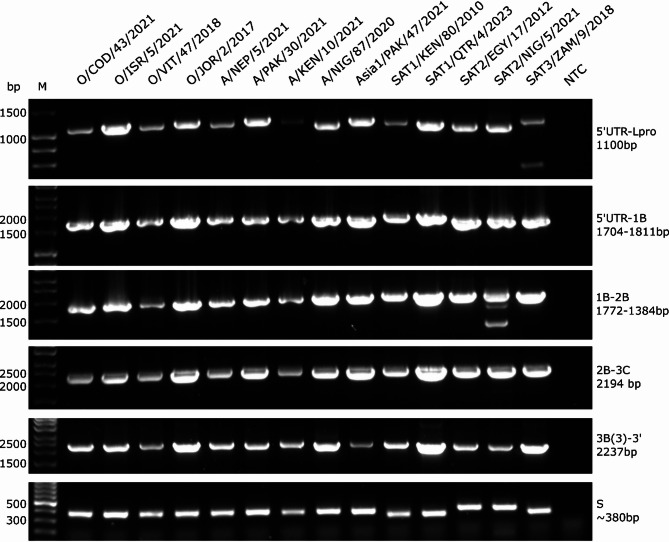
Gel electrophoresis of RT-PCR products generated for every serotype except C using the L_scheme amplification strategy. To the right-hand side of each gel the genomic regions which each product spans with the anticipated size underneath in base pairs (bp) is provided. M = Hyperladder 1KB molecular ladder or, for the S fragment, GeneRuler 100 bp (Thermo Fisher Scientific). Individual bands of the ladders are marked according to their size in base pairs (bp)

In addition to the S_scheme, a final arrangement of larger amplicons – termed the L_scheme - comprised one amplicon representing the S fragment and a further five overlapping amplicons encompassing the L fragment (Fig. 1B; Table 2). Primer combinations for the L_scheme were selected based upon the wider S_scheme testing but were also empirically tested to confirm performance (Fig. 2).

**Table 2:** L_scheme primer composition of pools A and B for the universal amplification of FMDV

Pool	Primer	Target serotype(s)	Sequence 5’−3’
A	UNI_For_1.1.1	All	GGCCACGCGTCGACTAGTACCCCCCCCCCCCCCCCCCCYAAR
	UNI_For_1.3.1	All	GGCCACGCGTCGACTAGTACCCCCCCCCCCCCCCCCCCRYN
	UNI_For_1.4.1	All	GGCCACGCGTCGACTAGTACCCCCCCCCCCCCCCCCCCRRN
	UNI_For_1.5.1	All	GGCCACGCGTCGACTAGTACCCCCCCCCCCCCCCCCCCTYW
	UNI_For_1.6.1	All	GGCCACGCGTCGACTAGTACCCCCCCCCCCCCCCCCCCTRN
	O_UNI_For_6	O	CGGACGAACATGACRGCVCACAT
	A_UNI_For_6.1	A	AGAACMAACATGACWGCVCACAT
	As1_UNI_For_6	Asia 1	CGCACYAACATGACGGCBCACAT
	SAT1_UNI_For_6A	SAT1	ATRAACCCGCGYACCAACACCA
	SAT1_UNI_For_6B	SAT1	ACCAATTCCTCAAYCCRMGRACGAACAC
	SAT2_UNI_For_6.1	SAT2,3	TACCCRCACCAGTTCATYAACCC
	UNI_For_15.1	All	GAAGAARCCTGTCGCYTTGAARGTGA
	UNI_Rev_2.3	All	ACACCTCRCTGGGYCGYGARGC
	UNI_Rev_2.4	All	CAGATCTCGCTRGGGCGAGT
	UNI_Rev_9	All	GCYACAGCGGCCATRCAYGACA
	FMD_UNI_RT	All	TACAACGCCTGTAGCATTCCTTTTTTTTTTTTTTTTTTTTGRWWW
B	UNI_For_2	All	CRTGTGTGCRACCCCRGCAC
	UNI_For_10	All	CCAACCCTGGRCCCTTCTTYTT
	Sfrag_F.1	All	TTGAAAGGGGGYRYTAGGG
	O_A_UNI_Rev_5	O, A	GGGGCGATGTTGGCRTAVACCTTRAT
	As1_UNI_Rev_5.2	Asia 1	GGTGCTGCATTCATGTARACCTTRAT
	SAT1_UNI_Rev_5A	SAT1,3	GTYTTGGGGTCHGTGTTTTGGAA
	SAT1_UNI_Rev_5A.1	All	AAGTCTTCGGGTCGGTGTTTTGG
	SAT2_UNI_Rev_5	SAT2,3	GCRATGTTGGCGTACACCTCRAC
	UNI_Rev_14A	All	CAGCAGATGGCYACYGTCTTVCC
	UNI_Rev_14B	All	CAGCAGAGYGCAACBGTCTTMCC
	UNI_Rev_14C	All	CAGCAGAKYGCMACKGTCTTMCC
	Sfrag_R.1	All	GGGGGGGGGGTGAAAGGYRG

Each L_scheme primer set was able to amplify every FMDV isolate tested, including representatives of each currently circulating serotype (Fig. 2). The L_scheme was therefore selected for further development due to its comparative simplicity as well as its ability to amplify diverse FMDV isolates. Each primer pair was allocated to one of two multiplex pools of primers (Table 2). Each pool amplified three amplicons which, once mixed encompassed the entire genome (Fig. 1). The pooled L_scheme PCR products were in turn used as the input for the Oxford nanopore rapid barcoding kit prior to sequence generation.

### Sequencing evaluation

To evaluate the universal nature of the sequencing protocol, we amplified and sequenced the genomes of 30 FMDV isolates. The list of isolates included representatives of all serotypes (except serotype C) and included examples of 27 lineages currently circulating in the field (Table 3).

**Table 3:** Isolates used for L_scheme nanopore sequencing evaluation

Isolate	Serotype	Topotype	Lineage	Sub-lineage	VP1 identity (%)	Accession number
IRN/24/2015	A	ASIA	Iran-05	SIS-10	100	PV524752
NEP/5/2021	A	ASIA	G-VII		100	PV524754
PAK/1/2020	A	ASIA	Iran-05	SIS-13	100	PV524756
PAK/30/2021	A	ASIA	Iran-05	FAR-11	100	PV524757
VIT/19/2017	A	ASIA	Sea-97		100	PV524758
KEN/10/2021	A	AFRICA	G-I		100	PV524753
NIG/87/2020	A	AFRICA	G-IV		100	PV524755
PAK/47/2021	Asia1	ASIA	Sindh-08		99.68	PV524759
CAM/3/2018	O	ME-SA	PanAsia		100	PV524741
ISR/5/2021	O	ME-SA	PanAsia-2	QOM-15	100	PV524746
SRL/13/2019	O	ME-SA	Ind-2001	d	100	PV524749
JOR/2/2017	O	ME-SA	Ind-2001	e	100	PV524747
UAE/2/2021	O	ME-SA	PanAsia-2	ANT-10	100	PV524750
GHA/1/2016	O	WA			100	PV524744
COD/43/2021	O	EA-2			100	PV524742
PAT/8/2021	O	EA-3			100	PV524748
ETH/24/2019	O	EA-4			100	PV524743
HKN/1/2019	O	CATHAY			99.84	PV524745
VIT/47/2018	O	SEA	Mya-98		100	PV524751
KEN/80/2010	SAT1	I			100	PV524760
QTR/4/2023	SAT1	I			100	PV524917
TAN/27/2012	SAT1	I			100	PV524918
NMB/1/2020	SAT2	III			99.54	PV524765
ZAM/8/2021	SAT2	IV			100	PV524767
NIG/57/2020	SAT2	VII			100	PV524763
NIG/91/2020	SAT2	VII			100	PV524764
SUD/12/2017	SAT2	VII	Alx-12		100	PV524766
EGY/17/2012	SAT2	VII	Ghb-12		100	PV524761
NIG/5/2021	SAT2	VII	Lib-12		100	PV524762
ZAM/9/2018	SAT3	II			100	PV524768

**Fig. 3:**
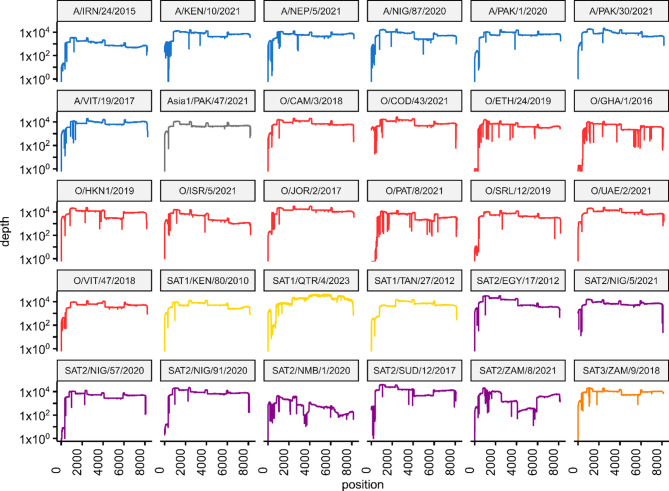
Coverage plots for FMDV isolates sequenced for the evaluation of the final arrangement of L_scheme primer pools. Six serotypes are represented encompassing 27 topotypes/lineages currently circulating globally

Every isolate yielded amplicons that could be sequenced using the MinION. Sequences were generated across the entire L fragment and, in the majority of cases, the S fragment (Fig. 3). However, occasional failures were observed for the S fragment, with 6/30 samples trialled resulting in low levels of coverage. For example, the sequence data for O/ETH/24/2019 and O/GHA/1/2016 only contained one and two reads specific for the S fragment respectively, despite substantial coverage of the L fragment.

A consensus sequence was generated using the lineage-agnostic NanoFMDV pipeline for each isolate, and the VP1 encoding regions compared to those obtained by the established Sanger-based sequencing approaches [27]. 100% nucleotide identity was observed for 27 of the 30 isolates sequenced (Table 3). Two synonymous substitutions were present for Asia1/PAK/47/2021. A guanine to cytosine substitution in O/HKN/1/2019 resulted in a non-synonymous (T861S) amino acid change, although a mixture of the bases was observed in the BAM file. SAT2/NMB/1/2020 exhibited two nucleotide substitutions and the insertion of a thymine nucleotide; however, lower coverage across the VP1 coding region was observed for this isolate (Fig. 3).

The initial sequencing runs performed in this study used PCR products which had been cleaned using either GFX columns or AmpureXP beads. However, this clean-up process is expensive, laborious and previous studies have reported that the clean-up of the PCR reactions prior to library preparation is not always necessary [40]. To evaluate the necessity for PCR cleanup of the amplicons designed here, twelve samples from the validation panel were re-amplified in duplicate. One set of each pair was purified using AmpureXP beads as previously described, whereas the paired sample was diluted 1:10, with 10 µl of the dilution being used directly as the input for library generation.

**Fig. 4:**
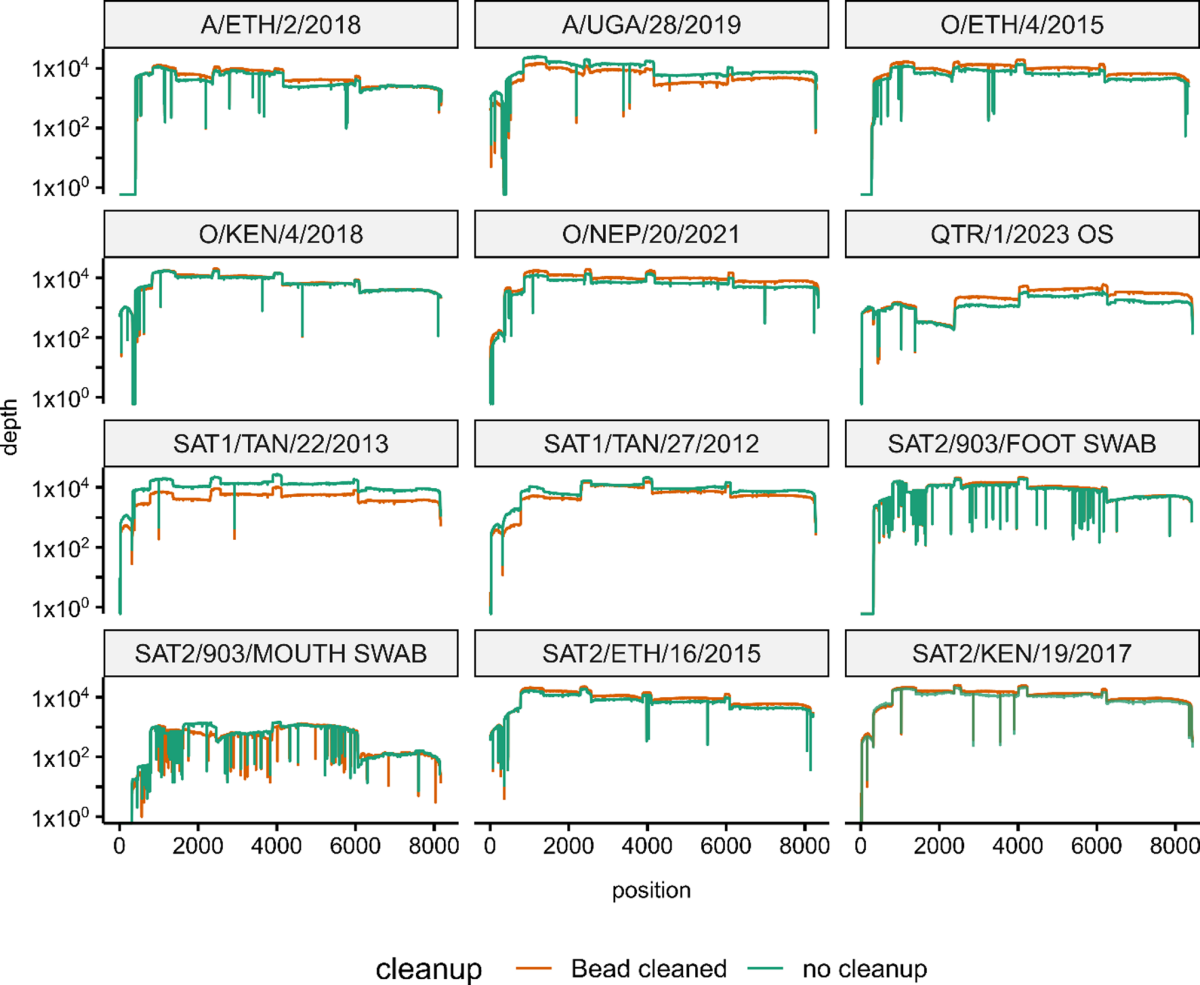
Coverage plots of sequence data obtained using PCR products either cleaned using AmpureXP beads (bead-cleaned; green) or diluted 1:10 in nuclease free water. PCR products were generated using the final arrangement of L_scheme primer pools. 903 refers to sequences derived from clinical swabs from an animal experimentally infected with SAT2/JOR/19/2023

The resulting data showed that the inclusion of a cleanup step had no impact upon the subsequent breadth and depth achieved (Fig. 4). One sample (SAT2/903/MOUTH SWAB) achieved a lower depth of coverage relative to the remaining samples, including the failure of the S fragment (Fig. 4). However, this deficiency applied to both the purified and unpurified PCR products implying that this is a PCR artefact, and still resulted in > 96% of every genomic position being covered by at least 10 reads. Therefore, it was concluded that PCR product cleanup is not required.

### In -country deployment of nanopore sequencing in Uganda

To demonstrate the utility of nanopore sequencing in an endemic setting where laboratory conditions are often limited, the initial S_scheme and L_scheme primer mixes were trialled in parallel in Uganda, a FMDV endemic nation within FMD endemic Pool 4 [3]. To reflect the limited capacity typical of laboratories in many endemic countries, we performed the RT as well as the PCR of some of the samples using a portable PCR machine (miniPCR bio™). Furthermore, we used a minicentrifuge and the easily portable Quantus fluorometer (Promega) for the quantification of DNA.

**Fig. 5:**
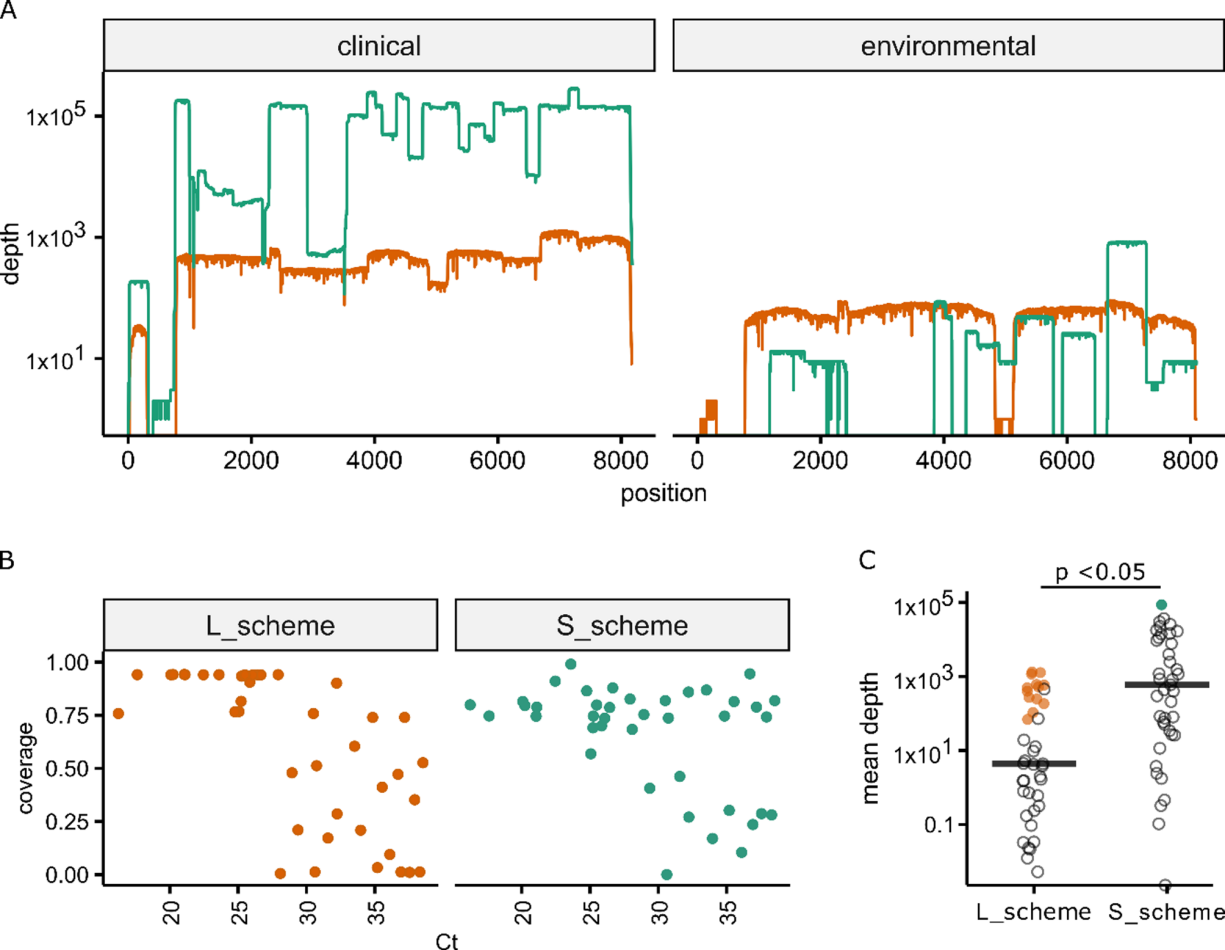
Performance of nanopore sequencing on FMDV positive samples collected in Uganda. (**A**) R epresentative coverage plots of genome sequences recovered from clinical (left) and environmental swabs. The orange trace represents the L_scheme and the green trace represents the S_scheme. (**B**) Scatter plots for the L (left) and S (right) primer schemes showing the proportion of the genome covered relative to real-time RT-PCR Ct value. (**C**) A comparison of the average depth at each nucleotide achieved using the L_scheme and S_scheme amplification strategies. Filled points represent samples resulting in near complete genomes

Both the S_scheme and L_scheme achieved near complete genomes (Fig. 5A and Additional files 3–4). However, the L_scheme outperformed the S_scheme, with the L_scheme/rapid barcoding approach resulting in 15 near complete genomes (median 94.3% of the reference genome covered) from samples with Ct values with a median Ct value of 23.6 (range 17.62–27.93) at an average depth of 518. While less consistent in generating complete genomes (Fig. 5B), the depth of coverage was on average greater when using the S_scheme (Fig. 5C). Whilst considerable variation in coverage depth was observed, for those samples generating near-full-length sequences, the depth was always sufficiently high to generate an accurate consensus (Fig. 5C, filled points). The most closely related genome to those obtained using the nanopore methodology was in every case SAT2/TAN/5/2012 (Accession number KM268900, 94.7–94.9% nucleotide identity). The amplification and sequencing of FMDV genomes was most successful (in terms of coverage) when using clinical samples. However, a large proportion of genome was sequenced using environmental swabs collected from a stick and a rope (Additional file 5).

**Fig. 6:**
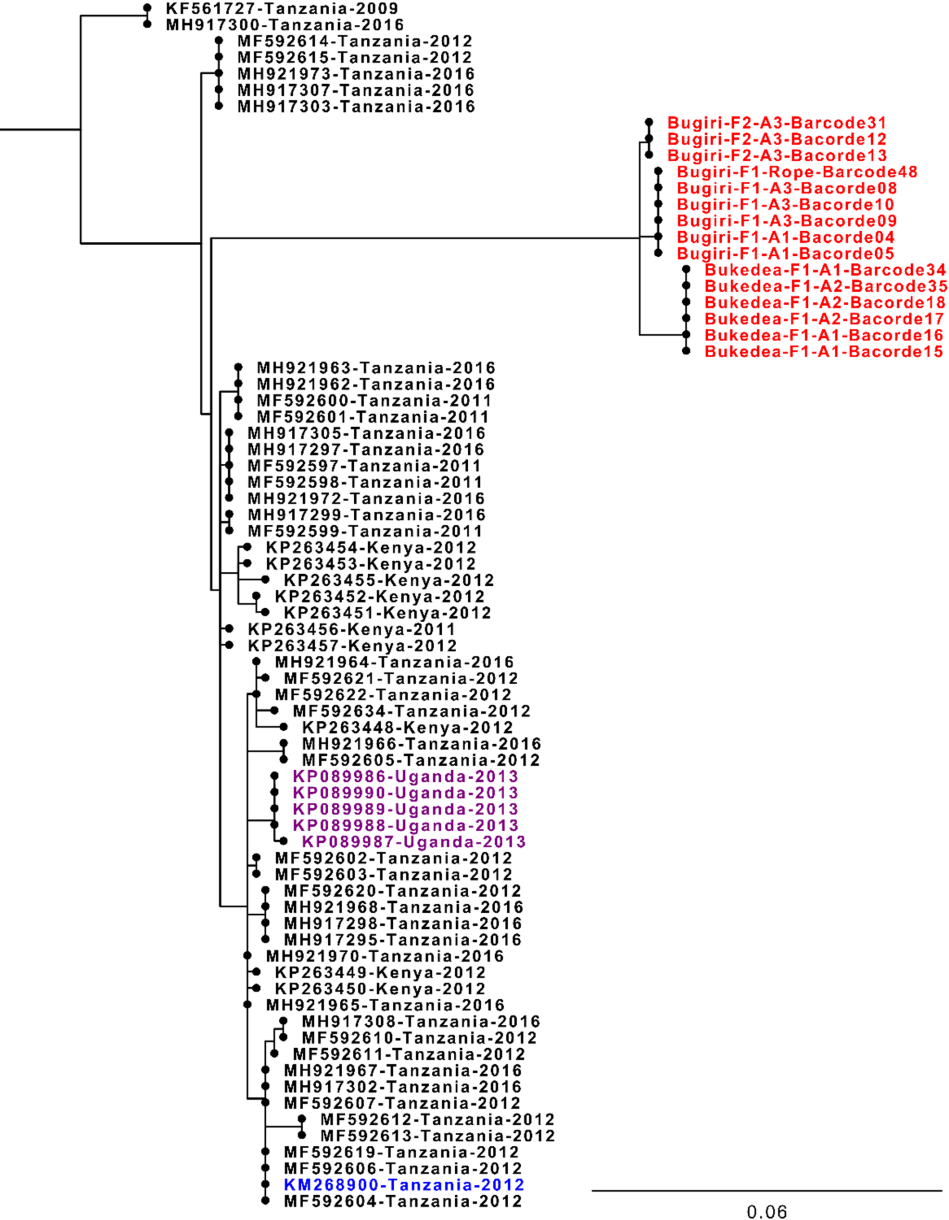
FMDV SAT2/IV VP1 phylogenetic tree incorporating the Ugandan sample VP1 sequences. VP1 sequences from Ugandan samples (highlighted in red) were labelled according to their District, Farm (F), Animal (A), and Barcode number. The VP1 from the closest existing complete genome sequence (Tanzania, 2012) is highlighted in blue, whilst existing SAT2/IV VP1 sequences from Uganda on GenBank are highlighted in purple; all other SAT/IV sequences are shown in black. This is a cropped version of the full VP1 phylogenetic tree, only displaying the cluster containing the Ugandan sample sequences generated in this study; the complete tree is available as Additional file 6. SAT2/I and SAT2/III lineage sequences were used as an outgroup

**Fig. 7:**
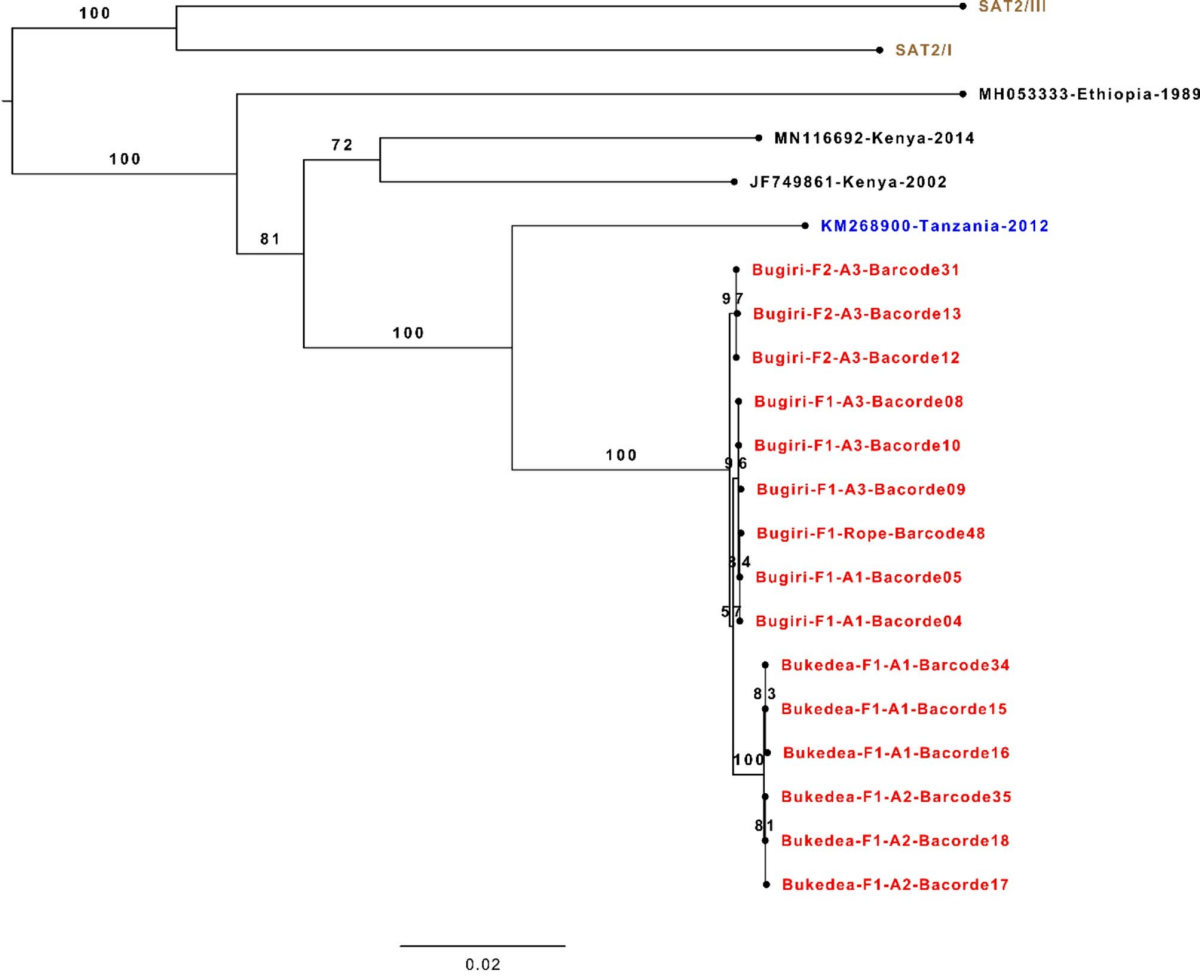
FMDV SAT2/IV complete genome phylogenetic tree incorporating the Ugandan sample consensus genomes. Consensus genome sequences from Ugandan samples (highlighted in red) were labelled according to their District, Farm (F), Animal (A), and Barcode number. The closest existing genome sequence (Tanzania, 2012) is highlighted in blue whilst other SAT2/IV complete genome sequences available from GenBank are shown in black. SAT2/I and SAT2/III lineage sequences (highlighted in brown) were used as an outgroup. Bootstrap values are shown next to their corresponding branches

Phylogenetic reconstruction of publicly available VP1 sequences retrieved from FMDbase (https://www.fmdbase.org) revealed that Ugandan viruses were most closely related to viruses collected from Tanzania and Kenya between 2011 and 2016, whereas the most recent Ugandan SAT2/IV sequences were collected in 2013 (Fig. 6). Among the sequences generated in this study, sequences clustered at both farm and district levels (Fig. 6). In contrast, phylogenetic links between individual animals sampled on each farm were resolved using sequences of the full-polyprotein coding region (Fig. 7).

## Discussion

It is well established that multiple serotypes and topotypes of FMDV often co-circulate within the regional endemic pools, where inter-pool movements of FMDV lineages can further complicate the epidemiology of the disease [6, 41]. Tools are urgently needed to improve the surveillance of FMD in these settings to understand the burden of disease and regional risk pathways. Furthermore, rapid identification of field FMD viruses is critical when there are new incursions of FMDV into FMD-free countries [42]. In view of the genomic diversity of FMDV, universal sequencing protocols are anticipated to provide capability for endemic and epidemic scenarios where a wide range of virus serotype and topotypes may need to be characterised.

Primer designs for universal genome amplification strategies have previously been published [43]. However, the necessity to locate primer binding sites within relatively restricted regions to generate approximately equally sized amplicons for the S_scheme necessitated a re-appraisal of all available primer sequences. Aligning primers to 1682 FMDV complete genomes enabled the rational selection of existing primers, as well as the design of new primer sequences that would be predicted to amplify all FMDV sequences. Whilst focusing upon currently circulating viruses may have resulted in a more streamlined set of primers appropriate for the current epidemiological situation, the use of historical sequences has instead allowed the identification of primer footprints that have not changed over several decades of evolution, providing greater confidence that these targets are highly conserved and unlikely to change. Furthermore, the design of primers capable of amplifying historical samples means that the schemes are appropriate for retrospective analyses of viruses held in laboratory archives.

The quality of data obtained using nanopore-based sequencing continues to improve as the chemistry and base calling models evolve [44]. We observed that using the super-high accuracy method greatly enhanced the accuracy of the resulting sequences. In common with many forms of HTS, homopolymeric regions often proved to be the areas that were most difficult to resolve. Insertions or deletions of nucleotides (indels) can be resolved manually for protein coding regions where amino acid conservation is required. However, in non-coding regions, where indels are frequently observed with FMDV [45–47], base-calling errors could influence the accuracy of the consensus genomic sequence.

The DNA PCR products used in the initial library preparations for this project (including in Uganda) were purified using GFX spin columns. Subsequently, we showed that the extent of data generated was not affected by the omission of this cleanup step. Whilst the exclusion of a cleanup step needs further evaluation, for example its impact upon sensitivity, it results in substantial savings in terms of both time and money, and is a finding in line with Freed e*t al* [40]. It should be noted that the validation undertaken here used only SuperFiII Green mastermix and it remains possible that other mastermixes may be inhibitory to downstream library generation. In addition, not all library preparation kits may tolerate the omission of a purification step. Preliminary results suggest that Q5 polymerase (New England Biolabs) is also suitable for use in this protocol, although diluting the PCR products 1:2 rather than 1:10 (data not shown). Similarly, barcoding alongside the ability to wash and reuse flow cells makes nanopore sequencing more cost effective and particularly attractive for use in regions where support for more established workflows is limited [38]. A previous study sequencing SARS-CoV-2 calculated that genomes could be sequenced at low cost using nanopore technology (at the time of publication £10/sample). The most efficient manner in which to reduce costs was found to be by multiplexing the samples and by reusing washed flowcells [48]. However, when reusing flowcells, the selection of barcodes needs to be carefully considered due to the possibility of carry-over between runs.

The coverage obtained for the S fragment was not always consistent. The occasional failure to obtain sequence for the S fragment may be due to weak PCR amplification as frequently observed via gel electrophoresis. An improvement to this method would therefore be to increase the efficiency of the S fragment within a multiplex format, although this is challenging due to the downstream flanking poly (C) tract. Alternatively, a three-reaction format could be used with a reaction dedicated to amplifying the S fragment. An alternative explanation is the fact that the rapid barcoding chemistry is based upon the random insertion of barcodes. The S fragment amplicon is ~ 380 bp, thus there are fewer positions at which a barcode can successfully be inserted and still result in a read longer than the required 200 bp (Oxford Nanopore Technologies, personal communication).

A distinct advantage of using a larger fragment size is the ability to use the rapid barcoding kit as well as the requirement for fewer primers per reaction. Indeed, despite a larger amplicon size, the L_scheme outperformed the S_scheme when tested using clinical and environmental swabs collected in Uganda. This result suggests that there is scope for improvement in the efficiency of the S_scheme PCRs. Despite the clear advantages of the L_scheme when sequencing FMDV, in situations requiring greater analytical sensitivity, e.g. wastewater analysis, an optimised primer scheme based upon short fragments has merit.

Trialling the sequencing approaches in Uganda highlighted the benefits of using swabs derived from animals exhibiting clinical signs relative to environmental swabs. Genomes were also generated using swabs collected from the lesions of experimentally infected cattle, further demonstrating the suitability of clinical lesion swabs as a sample matrix. It is anticipated that these results can be explained by the higher titre of FMD virus present in clinical samples, as well as difficulties to sequence degraded RNA in environmental swabs due to environmental factors such as heat and ultraviolet light exposure [49]. However, the sequencing of FMDV from environmental samples complements the data obtained from clinical samples and can contribute to uncovering active transmission chains during outbreaks [49, 50]. Furthermore, the integration of field-appropriate methods in resource-limited settings significantly enhances the scalability of FMDV sequencing in endemic areas. The successful generation of sequence data in Uganda demonstrated that this method is possible to use with limited equipment and resources. Nevertheless, the development of lyophilised/dried reagents with stability at room temperature would be hugely beneficial in locations with a propensity for power supplies to be interrupted.

The generation of near complete genomes enabled phylogenetic trees to be constructed using the complete coding sequence of the virus. Using a greater proportion of the genome enabled individual animals to be distinguished on each farm. Thus, these data further demonstrate the power of complete genome sequence analyses when informing epidemiological studies. In particular, reconstructing the path of infection from animal-to-animal and farm-to-farm through complete genome sequencing complements network analyses of livestock movements via contact tracing and trade data. This approach can be integrated with other types of epidemiological data for accurately parameterizing dynamic models in complex eco-epidemiological systems involving multiple hosts, viral evolution and environmental persistence.

The pipeline developed here was entirely agnostic with regards to FMDV serotype and lineage, and correctly assembled the genomes of every virus trialled. However, whilst infrequent, FMDV co-infections do occur, most notably the SAT serotypes which persist in African buffalo. Similarly, recombination readily occurs between co-infected viruses, suggesting that a pipeline reliant upon reference assembly may struggle to generate genome length sequences of recombinant viruses. Further work is required to fully establish the ability of the pipeline to detect the presence of, and assemble the genomes of, viruses derived from mixed infections.

In summary, we have developed a universal RT-PCR and sequencing approach capable of generating complete FMDV genomes. The method, in combination with the NanoFMDV pipeline, was successfully trialled in Uganda and represents a route to straightforward and economical sequencing of FMDV genomes in laboratory settings where NGS infrastructure is lacking.

## Methods

### Amplicon design

Primers predicted to encompass the full extent of FMDV diversity were designed using the publicly available sequences hosted in FMDbase. Complete FMDV genome sequences in FMDbase were downloaded and aligned using MAFFT v7.453. Many of the multiplex ‘ARTIC’-like approaches for sequencing small viral genomes utilise small amplicons approximately 400 bp in length. We therefore developed a similar approach, termed S_scheme, using two PCR reactions to amplify 20 overlapping tiles. However, the nucleotide diversity evident across the FMDV genome necessitated flexibility in the precise location of primer footprints. An amplicon strategy utilising fewer, but larger PCR amplicons (termed L_scheme, analogous to the ‘Midnight’ protocol used for SARS-CoV-2 [40]) divided across three PCR reactions was trialled in parallel with the S_scheme. In addition to requiring fewer PCR amplicons and therefore fewer conserved primer sites, the L_scheme utilised the ONT rapid barcoding kit, which is simpler, quicker and cheaper [40]. An initial screen was undertaken to appraise previously published primer sites, for example those described in Knowles et al. [27] and Dill et al. [43], for their suitability in terms of i) cross-reactivity among serotypes and topotypes and ii) position within the genome. In certain cases, it was possible to modify some of the published primers, for example the FMD-L137F primer from Dill et al. [43] was adjusted in terms of both its position and its incorporation of redundant bases. However, in the majority of cases, the necessity for specific regions to be targeted required the design of new primers with broad reactivity across the FMDV species. Software for the design of tiled amplicons, for example PrimalScheme, have previously been developed. However, the extent of genomic diversity tolerated by the original PrimalScheme algorithm precluded its use in the development of a pan-FMDV primer scheme. Therefore, primer locations were identified manually by visual inspection of alignments. The level of cross-reactivity of prospective primers was assessed using a custom R script (Additional files 7–8), and the melting temperatures predicted using OligoEvaluator™ (Sigma-Aldrich).

With certain exceptions, for example A, the P2 and P3 regions of the FMDV genome are relatively conserved (68–91% nucleotide identity [51]), and a limited set of oligos was therefore sufficient to match the majority of the sequences. In contrast to the P2 and P3 regions, the P1 region of the FMDV ORF is highly diverse, with particularly high levels of sequence variability (48–75% nucleotide identity [51]), observed across the 1B, 1C, and 1D regions (encoding VP2, VP3 and VP1 respectively). A previous route to avoid the issue of variability in P1 has been to generate a PCR amplicon which spans the entire capsid coding region, with primers located in Lpro and 2A/2B [29, 33]. However, in order to achieve efficient multiplex amplification of the entire genome (most notably in the case of the S_scheme), we aimed to amplify amplicons of approximately equal size, resulting in some amplicons requiring multiple primers targeting the same (or a nearby) footprints (median = 3.5 primers per amplicon; range = 2–13). This was most evident in amplicon 7, which included seven sense primers and six antisense primers (Additional file 9; Table S3). Furthermore, certain sites within the capsid coding region required multiple primers within a serotype, most notably for serotypes SAT1 and SAT2, where the sequences were sufficiently distinct between east and west African clades (Table 2). In situations where the extent of diversity would require a multitude of primers, we adopted a rational approach by prioritising currently circulating lineages for successful amplification as they are the most likely sequences to be encountered. Separate alignments of the genomes for each serotype were prepared when designing primer schemes for footprints within regions encoding structural proteins.

**Fig. 8:**
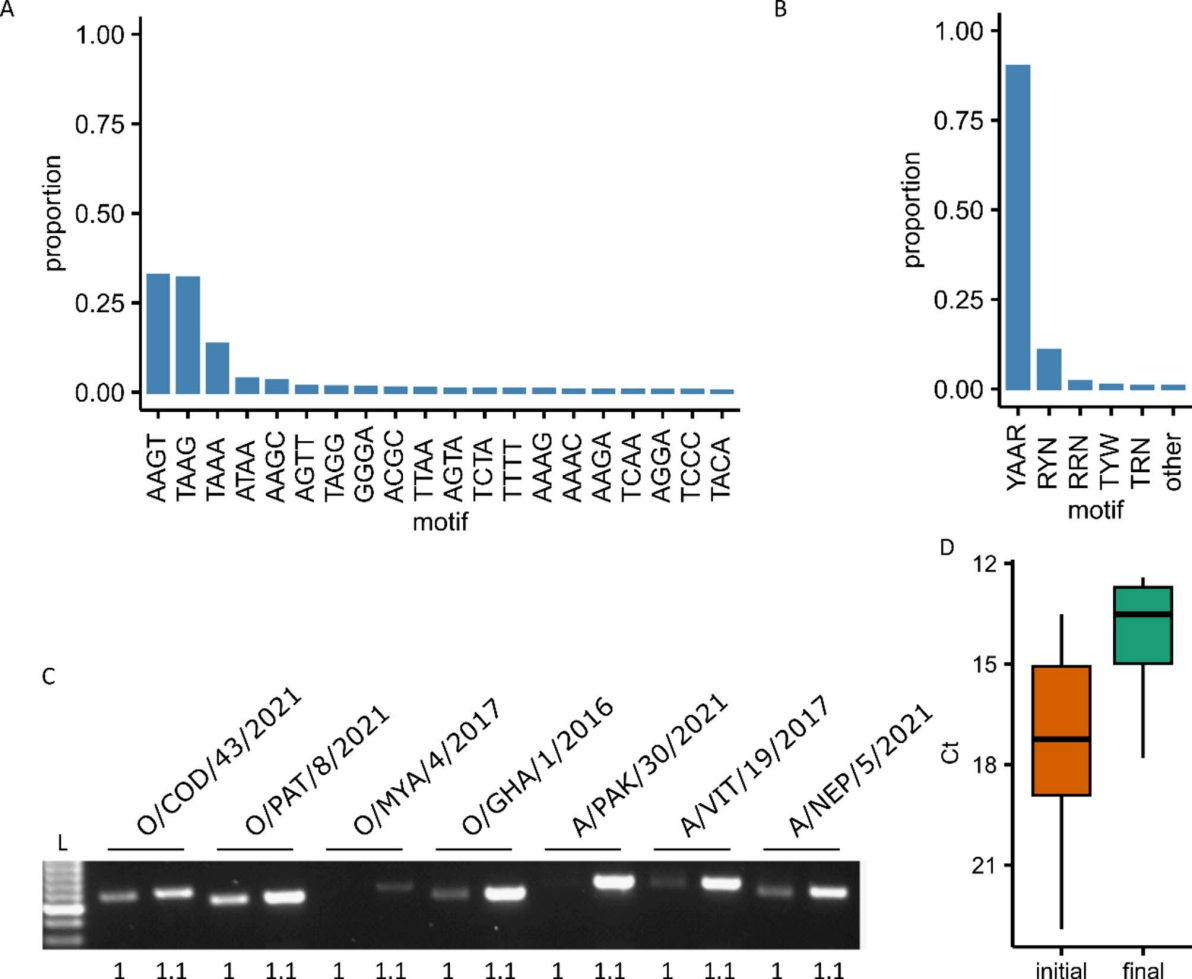
Optimisation of the UNI_For_1 primer region. (**A**) Proportion of each four-nucleotide sequence observed immediately following the poly(C) tract. Sequences were tallied from 1475 representative sequences. (**B**) The proportion of sequences encompassed by each of the UNI_For_1 primer variations. Only the 3’-most nucleotides specific to each primer are presented. ‘other’ equates to four nucleotide sequences not covered by any of the five primer variations (*n* = 6). (**C**) Agarose gel and semi-quantitation (**D**) of PCR products generated using the initial (1) and final (1.1) primer designs for UNI_For_1. L: molecular weight ladder (GeneRuler 100 bp, Thermo Fisher Scientific Scientific)

The region immediately following the poly(C) region of the FMDV region contains multiple pseudoknots and is extremely variable [52]. We countered this by using primers comprising a string of cytosines followed by a short sequence of FMDV L fragment sequence. The initial four bases immediately following the poly(C) were extracted and tabulated revealing a wide variety of possible combinations (Fig. 8A). Although variable, it was possible to allocate the majority of the sequences to one of just five consensus sequences (Fig. 8B). Five sense primers were therefore used for the amplicon immediately following the poly(C) region. Each version of the sense primer for the amplicon following the poly(C) was modified by extending the primer in the 5’ direction as initial attempts without this extension were variable (Fig. 8C). The 5’ extensions were shown to increase both reliability (Fig. 8C) as well as efficiency (Fig. 8D).

We incorporated these modified UNI_For_1 primers into a final L_scheme arrangement of two pan-FMDV multiplex PCRs amplifying long fragments (range 1109–2237 bp) and the S fragment (~ 380 bp) (Fig. 1B; Table 2).

**Fig. 9:**
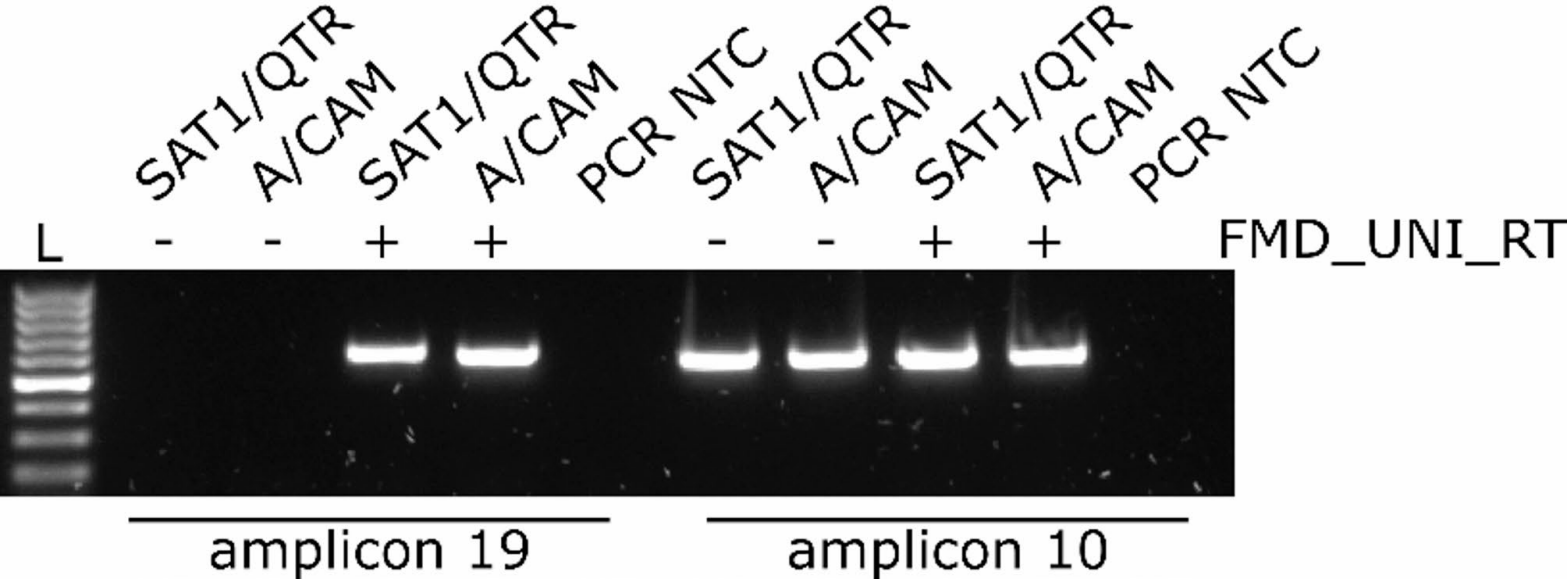
Agarose gel demonstrating the requirement for FMD_UNI_RT inclusion in the reverse transcription (RT) step. The 3’most amplicon (amplicon 19) was amplified from SAT1/QTR/4/2023 and A/CAM/5/2015 cDNAs prepared with (+) or without (-) FMD_UNI_RT in the RT reaction. A control amplicon (amplicon 10) amplifying the initial part of P2 not dependent upon the inclusion of FMD_UNI_RT. NTC = no template control. L: molecular weight ladder (GeneRuler 100 bp, Thermo Fisher Scientific Scientific)

The extreme 3’ of the 3’UTR immediately preceding the poly(A) tail is highly diverse, with insufficient homology to design primers capable of amplifying diverse viruses. We resolved this by using FMD_UNI_RT, which incorporates a 5’ extension of the poly-T stretch with a M13 primer sequence, as well as a short, redundant FMDV-specific consensus sequence (GRWWW) to help target and specifically anchor the primer to FMDV genomes. Importantly, this primer needed to be added to the reverse transcription reaction (Fig. 9).

Redundant bases were judiciously included to increase the likelihood that diverse FMDV sequences would be amplified, with a focus upon reducing template-primer mismatches towards the 3’ end of the primer. The primer extends from this end and previous studies have indicated that mismatches in the five 3’-most nucleotides have a disproportionately greater impact upon the success of amplification relative to the 5’ end of a primer.

Notably, serotype C viruses were not considered a priority for the schemes designed here as isolates of this serotype have not been observed in the field since 2004, leading to suggestions that serotype C is extinct in the wild [2]. However, alignment of the primer sequences against serotype C genomes (*n* = 40), suggests that serotype C isolates would likely still be amplified using the selected primers (Additional file 10).

### Viruses and samples

Samples (*n* = 53) used for the initial development and evaluation comprised RNA derived from viruses isolated from samples submitted to the WRLFMD at The Pirbright Institute (Tables 1 and 3 and Additional file 11).

### Clinical and environmental samples

RNA samples were derived from swab samples collected from farms with ongoing outbreaks of FMDV in three districts in Uganda (Budaka, Bugiri and Bukedea). 4N6FLOQ™ swabs (Thermo Fisher Scientific) were used to swab possible lesions present on either the feet or mouth (‘clinical’ swabs, *n* = 40) and were placed into 1 mL PBS. Lesion swabs were collected from animals at different clinical stages of infection including at > 10 days representing almost healed lesions. Environmental samples (*n* = 14) were collected as previously described [53, 54] at farms on which infected cattle were present. The Ugandan National Council of Science and Technology (UNCST) and the University of Edinburgh Animal Welfare and Ethical Review Board (AWERB) committee gave approval for both animal sampling (OS5-21) and human questionnaires. A 150 µL aliquot of the swab eluate was added to 337.5 µL of MagMax core lysis buffer (Thermo Fisher Scientific) and chilled whilst in the field. Upon return to the laboratory, samples in lysis buffer were stored at −80 °C until extraction. Swabs were first assessed for FMD presence using real-time RT-PCR as previously described [55]. Fifty-four swab samples positive for FMDV by real-time RT-PCR (Ct range 16.23–43.65, mean 30.53) were subjected to sequencing using both the S_scheme and L_scheme protocols.

Additional lesion swab samples were collected from cattle experimentally infected with FMDV at the Pirbright Institute. Animals used in this study were sourced from a commercial farm with defined health status. Prior to inoculation, animals were housed in groups to acclimatise for seven days prior to inoculation. Animals were sedated prior to FMDV challenge by administration of 0.3 mg/kg of xylazine via the intramuscular route. Cattle were placed into sternal recumbency, inoculated with FMDV (1 × 10^5^ TCID_50_ into the dorsal epithelium of the tongue), and then the sedation reversed by administering 0.1 mg/kg atipamezole. At the time of euthanasia, animals were secured and 140 mg/kg of sodium pentobarbital administered via the intravenous route. All experimental procedures were conducted in accordance with the Pirbright Institute’s AWERB, and the Home Office Animals (scientific procedures) Act 1986.

### RNA extraction

#### Virus isolates

Viral RNA was isolated from 0.5 mL cell culture supernatants maintaining the necessary ratios of lysis buffer to sample. RNA was extracted using the RNeasy mini kit columns (Qiagen) according to the manufacturer’s conditions. RNA samples were eluted from the columns in 50 µL nuclease free water and stored at −80 °C.

#### Swab eluates

Viral RNA was isolated manually using a magnetic bead approach using the MagMax core reagents (Thermo Fisher Scientific). Briefly, 7.5 µL of beads/proteinase K mix (2:1 ratio) was added to 125 µL of lysis buffer/sample and mixed thoroughly followed by the addition of 87.5 µL of binding solution. 125 µL of wash 1 solution was added and mixed thoroughly. The beads were captured with a magnet, the supernatant discarded and washed with 125 µL of wash 2. Following capture and discard of the wash supernatant, the RNA was eluted from the beads in 90 µL of elution buffer and stored at −80 °C.

### Reverse transcription

Reverse transcription was performed using the LunaScript SuperMix (New England Biolabs). Although LunaScript SuperMix already contains oligo dT and random hexamers, reactions were performed with the addition of the FMD_UNI_RT primer. Briefly, RT mixes comprised 2 µL Lunascript SuperMix (5X), 0.5 µL FMD_UNI_RT (50 µM stock) and 7.5 µL sample RNA. The components were mixed and incubated for two minutes at 25 °C, 10 min at 55 °C and 1 min at 95 °C. Following incubation, cDNA samples were either used immediately or stored at −20 °C. cDNA samples were assessed for FMDV titre using real-time PCR and, on average had a Ct of 20.26 (Additional file 11).

### PCR amplification and cleanup of sequencing template

All conventional PCR reactions used SuperFiII Green PCR mix (Thermo Fisher Scientific) in a PCR volume of 10 µL. When individual amplicons were amplified, 5 pmols of each primer were used. Multiplex reactions were performed by first mixing equal volumes (except for S_Frag_F.1 and R.1, for which double the volume was used), of each forward and reverse stock primer (100 µM) in a single tube followed by a 1:10 dilution to make a working stock, of which 4 µL was used in the final 10 µL PCR reaction. Reaction volumes were adjusted to 9 µL to which 1 µL of cDNA was added. S_scheme PCR cycling was as follows: 98 °C for 1 min (x1), 30 cycles of 98 °C for 10 s, 60 °C–10 s and 72 °C for 15 s, followed by a final extension of 72 °C for 5 min. PCR cycling for the larger L_scheme fragments was as follows: 98 °C for 1 min (x1), 35 cycles of 98 °C for 10 s, 60 °C–15 s and 72 °C for 3 min, followed by a final extension of 72 °C for 5 min. PCR products were analysed using 1% agarose gels stained with SYBR Safe gel stain (Thermo Fisher Scientific) and were purified using either the GFX PCR DNA and Gel Band Purification kit (Cytivia) or AmpureXP Beads according to the manufacturer’s instructions, eluting the DNA in 50 or 15 µL water respectively.

### Real-time RT-PCR

#### Hydrolysis probe based real-time PCR

RNA isolated from swab samples was tested for FMDV presence using the Callahan 3D PCR primers and probe targeting the viral 3D RNA polymerase [56]. The real-time RT-PCR reactions were run as previously described [55] using Toughmix RT-PCR mix (Quantabio, VWR) or Luna Universal qPCR probe mix on a QuantStudio 5 Real-Time PCR machine (Applied Biosystems).

#### SYBR-based real-time PCR

A universal PCR mix (Luna Universal qPCR, New England Biolabs) was used to semi-quantitate the efficiency of amplification. Half reactions comprising 5 µL 2x Luna Universal qPCR mix, 2.5 pmols each primer, 3.5 µL nuclease free water and 1 µL PCR product (diluted 1:10,000). To measure specific amplicons the reverse primer of the amplicon of interest was used in combination with the forward primer of the downstream amplicon. Reactions were cycled in a QuantStudio 5 Real-Time PCR machine using the fast profile followed by a melt curve step.

### Nanopore sequencing

S_scheme products were sequenced using the Oxford Nanopore Technologies (ONT) native barcoding kit (NBD) and the L_scheme products were sequenced using the ONT rapid barcoding kit (RBK). Initial trials used SQK-NBD-110.96/SQK-RBK-110.96 chemistry and R9.4.1 flow cells. However, the final evaluation as well as sequencing in Uganda used the most recent chemistries (kits SQK-NBD114.96 and SQK-RBK114.24 (Oxford Nanopore Technologies, Oxford, UK) for S_scheme and L_scheme fragments respectively) in conjunction with R10.4.1 flow cells (Oxford Nanopore Technologies). Nanopore sequencing of FMDV positive Ugandan swab samples was undertaken at Makerere University College of Veterinary Medicine, Animal Resources and Bio-Security (COVAB).

### Evaluation of nanopore sequencing

**Fig. 10:**
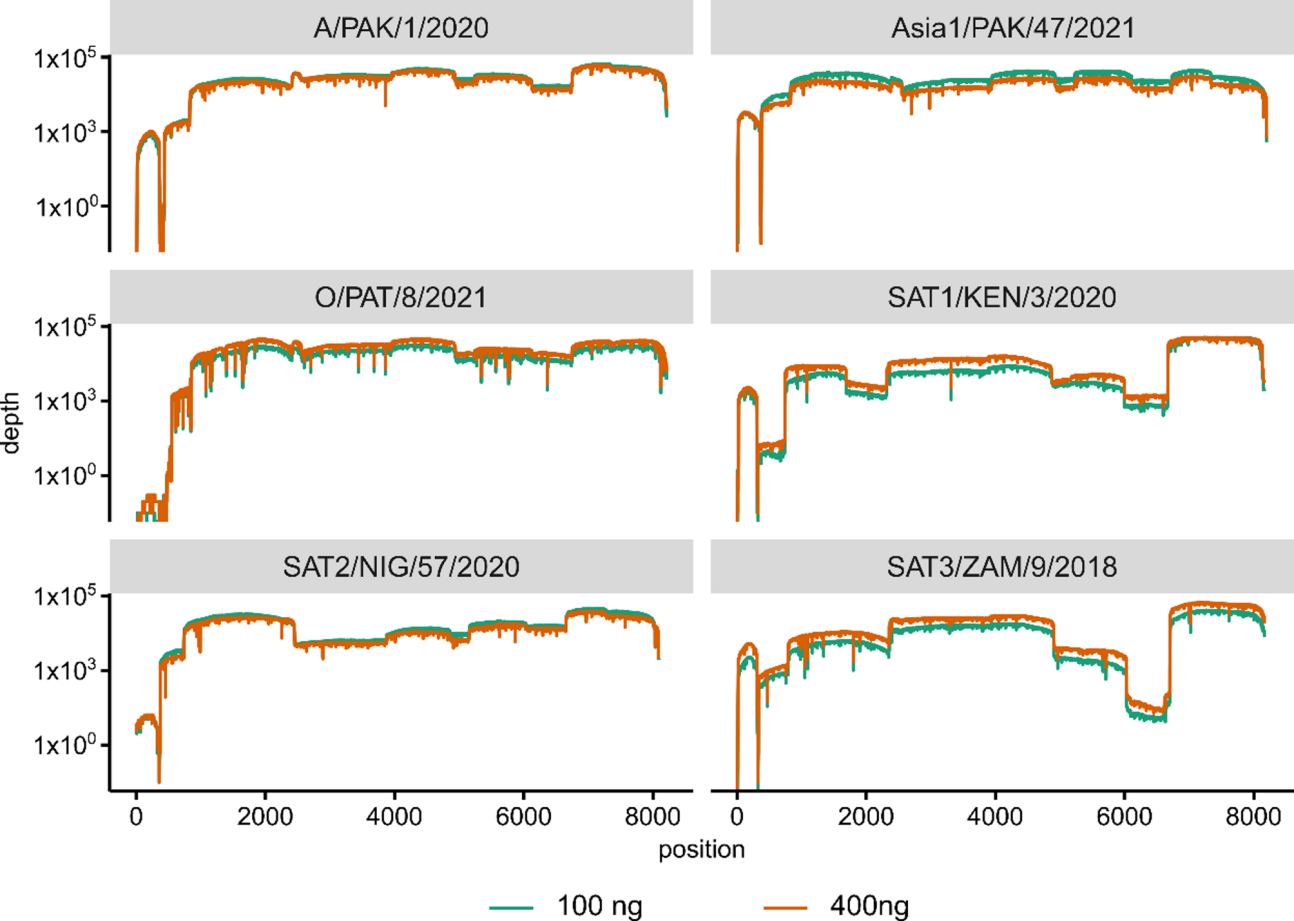
Impact of input mass on the coverage achieved across FMDV genomes. Representative isolates were used for serotypes O, A, Asia 1, SAT1, SAT2 and SAT3. Libraries were prepared using 100 (green trace) or 400 (orange trace) ng of input DNA. PCR products were generated using the L_scheme

Pilot sequencing runs utilised 400 ng of PCR product per sample to prepare libraries. To determine whether it would be possible to use a lower mass of input DNA, we performed sequencing reactions with a total of either 400 ng or 100 ng of PCR product. Reducing the mass of DNA had minimal effect regardless of serotype (Fig. 10). Although we successfully obtained genomes using significantly less DNA, subsequent libraries were prepared using (where quantified) 100 ng of DNA.

A target input mass for the SQK-NBD114.96 libraries was 70 ng whereas the target input for SQK-RBK114.24 libraries was 100ng. In situations where less than the target mass was achieved, library preparation continued with the maximum volume of product achievable (11.5 µl–10 µl for SQK-NBD114.96 and SQK-RBK114.24 respectively). Libraries were prepared according to the manufacturer’s instructions and included BSA in the flow cell priming mix. Flowcells were run using either a MinION Mk1B or Mk1C for 24–72 h. Raw data were basecalled using the super-high accuracy model within the MinKNOW software (MinKNOW version 24.06.5; Dorado version 7.4.12).

### Phylogenetic analysis

Both BLAST and FMDbase (https://www.fmdbase.org) were used to determine the FMDV serotype/topotype of the nanopore generated consensus sequences. Consensus sequences were aligned with all complete genomes of the identified SAT2/IV topotype available on GenBank (*n* = 4) with exemplar genome sequences representing the SAT2/I and SAT2/III topotype as an outgroup. Sequences were aligned using MAFFT v7.520, and a phylogenetic tree was constructed using IQ-TREE (v2.0.6) with 1000 bootstrap replicates; the Transversion Invariant Model TIM2 + F + I + G4 was identified as the best fit substitution model. Additionally, all available SAT2/IV sequences available on GenBank were aligned using MAFFT along with the nanopore generated consensus sequences and SAT2/I and SAT2/II outgroups. The corresponding VP1 section of the alignment was manually extracted (*n* = 204 GenBank VP1 sequences), and a phylogenetic tree was constructed using IQTREE-2 with 1000 bootstrap replicates; TIM2 + F + I + G4 was identified as the best fit substitution model.

### Bioinformatics pipeline

The bespoke bioinformatics pipeline is currently implemented via a collection of bash scripts, which call existing bioinformatics tools as well as custom Python (version 3.8.15) scripts (Additional file 12). The pipeline, referred to as NanoFMDV, along with the reference FMDV sequences, and usage instructions is publicly available at: https://github.com/rjorton/nanofmdv. The first step in the bioinformatics pipeline is to determine an appropriate FMDV reference sequence to use for read alignment. A database of 79 near complete FMDV genomes were downloaded from the FMDbase sequence database (https://www.fmdbase.org), representing approximately one per existing FMDV lineage; typically the FMDbase ‘RefSeq’ was selected for each lineage if available, unless a significantly longer genome sequence (ignoring N bases) was available. The VP1 sequence of each genome was extracted to create an equivalent FMDV-VP1 sequence data set. Each demultiplexed sample is analysed independently. After concatenating all of a sample’s FASTQ reads into a single file, minimap2 (version 2.24-r1122) [57] is used to align the reads to the FMDV-VP1 sequence collection. The VP1 reference sequence with the highest number of reads mapping to it is selected as the reference sequence for the sample, with minimap2 again used to align all the reads on to the reference FMDV genome corresponding to the selected VP1 sequence. As tiled amplicons are used, amplicon primer sequences must be trimmed before consensus sequence generation. A simple Python script, based on the previous GoPrime [58] methodology, is used to compare all the possible forward and reverse primers of each amplicon to the selected reference sequence to identify possible binding sites. A BED file is subsequently created representing the binding regions of each amplicon’s primers on the reference sequence. The samtools (version 1.16.1) [59] ampliconclip function is then used to clip primer sequences from the aligned reads within the BAM file using the previously created BED file, with a primer-clipped read FASTQ file also generated using the samtools fastq function. A preliminary consensus sequence is generated using the samtools consensus function. This preliminary consensus sequence, along with the primer-clipped read FASTQ file, is then used by the nanopore variant/consensus caller medaka (https://github.com/nanoporetech/medaka, version 1.7.2) to call a ‘polished’ FMDV consensus genome sequence (medaka has models to address nanopore associated errors such as those at homopolymers), the consensus sequence is also depth masked to mask positions with read coverage less than 10 with N bases. A final step in the pipeline, is to BLASTn [60] the final consensus sequence against a collection of 923 FMDV genome sequences to determine the closest sequence matches and their respective serotype/topotype. Assembly statistics were generated using samtools. Analyses were performed using R (version 4.4.0) [61]. An example fastq dataset has been submitted to the ncbi short read archive (SRA, accession number PRJNA1286760; https://www.ncbi.nlm.nih.gov/sra/PRJNA1286760) for SAT1/TAN/27/2012 which, when assembled should result in the same sequence as accession number MT602086.

## Supplementary Information

Supplementary Material 1.

Supplementary Material 2.

Supplementary Material 3.

Supplementary Material 4.

Supplementary Material 5.

Supplementary Material 6.

Supplementary Material 7.

Supplementary Material 8.

Supplementary Material 9.

Supplementary Material 10.

Supplementary Material 11.

Supplementary Material 12.

Supplementary Material 13.

Supplementary Material 14.

Supplementary Material 15

Supplementary Material 16.

Supplementary Material 17.

Supplementary Material 18.

## Data Availability

Sequence data generated during the validation of this method have been submitted to GenBank and are available under accession numbers PV524741- PV524918. Further sequences generated in Uganda have been submitted to GenBank and are available under accession numbers PV883408 - PV883420. A set of FASTQ files has been uploaded to the short read archive and are available under accession number PRJNA1286760.
