# Supplementary material for: Universal amplification and sequencing of foot-and-mouth disease virus complete genomes using nanopore technology
Source: BMC Genomics. 2025 Aug 22;26:770. doi: 10.1186/s12864-025-11938-7 (PMC12372193; doi:10.1186/s12864-025-11938-7)
Supplement: Supplementary file 1 — Supplementary Material 1. [file 12864_2025_11938_MOESM1_ESM.pdf]

**Table S2.** PCR outcome for initial S_scheme primer designs for each amplicon targeting a panel of Southeast Asian (pool 1) FMDV isolates.

| Virus isolate | Lineage | S | 1 | 2 | 3 | **4*** | **5** | **6** | **7** | **8** | **9** | 10 | 11 | 12 | 13 | 14 | 15 | 16 | 17 | 18 | 19 |
|---|---|---|---|---|---|---|---|---|---|---|---|---|---|---|---|---|---|---|---|---|---|
| VIT/1/2017 | O/SEA/Mya-98 | + | + | + | + | + | + | + | + | +/- | + | + | + | + | + | + | | + | + | + | + |
| VIT/1/2018 | O/ME-SA/PanAsia | +/- | + | + | + | + | + | + | + | + | + | + | + | + | + | + | + | + | + | + | + |
| VIT/11/2017 | A/ASIA/Sea-97 | + | + | + | + | +/- | + | | + | + | + | + | + | + | + | + | + | +/- | + | + | + |

*Bold typeface indicates amplicons for which at least one primer is located within the outer capsid coding genes (1B,1C and 1D).
