# Supplementary figures and images for: Universal amplification and sequencing of foot-and-mouth disease virus complete genomes using nanopore technology
Source: BMC Genomics. 2025 Aug 22;26:770. doi: 10.1186/s12864-025-11938-7 (PMC12372193; doi:10.1186/s12864-025-11938-7)

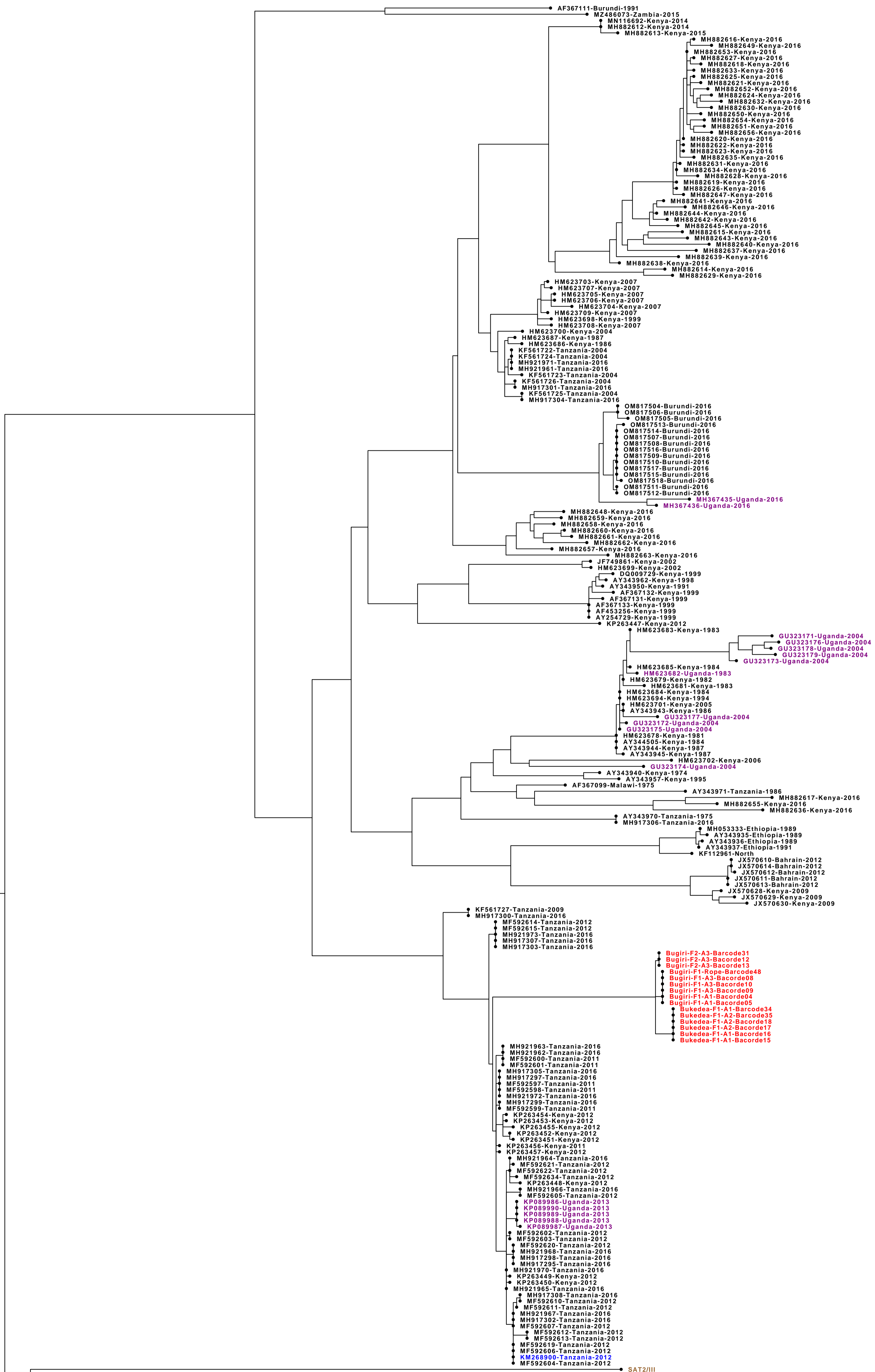

Supplement: Supplementary file 2 — Supplementary Material 2. [file 12864_2025_11938_MOESM2_ESM.pdf]

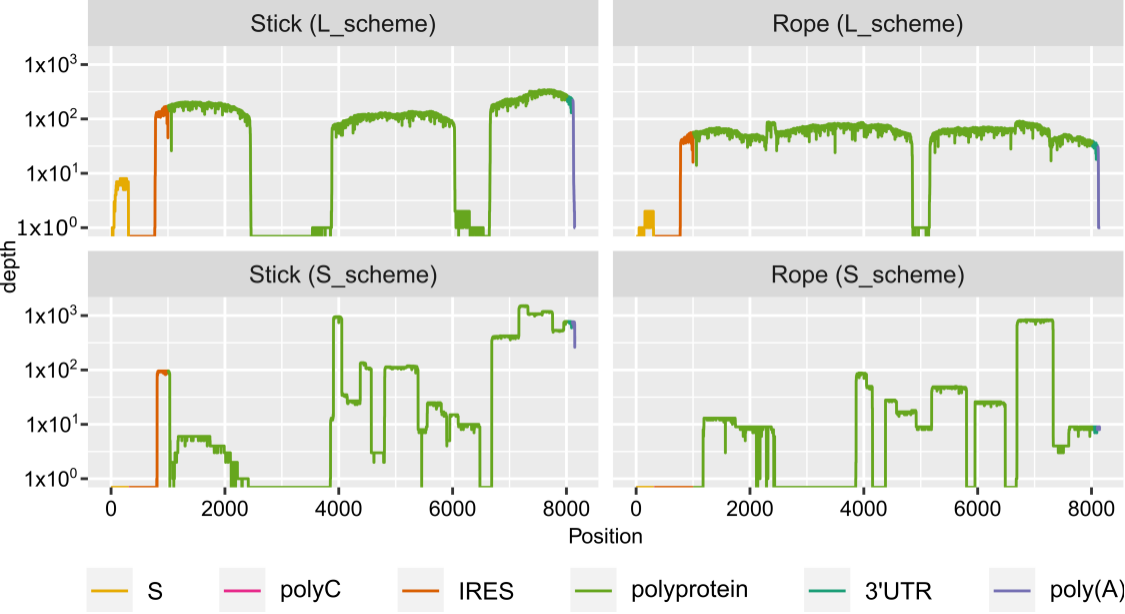

Supplement: Supplementary file 3 — Supplementary Material 3. [file 12864_2025_11938_MOESM3_ESM.pdf]

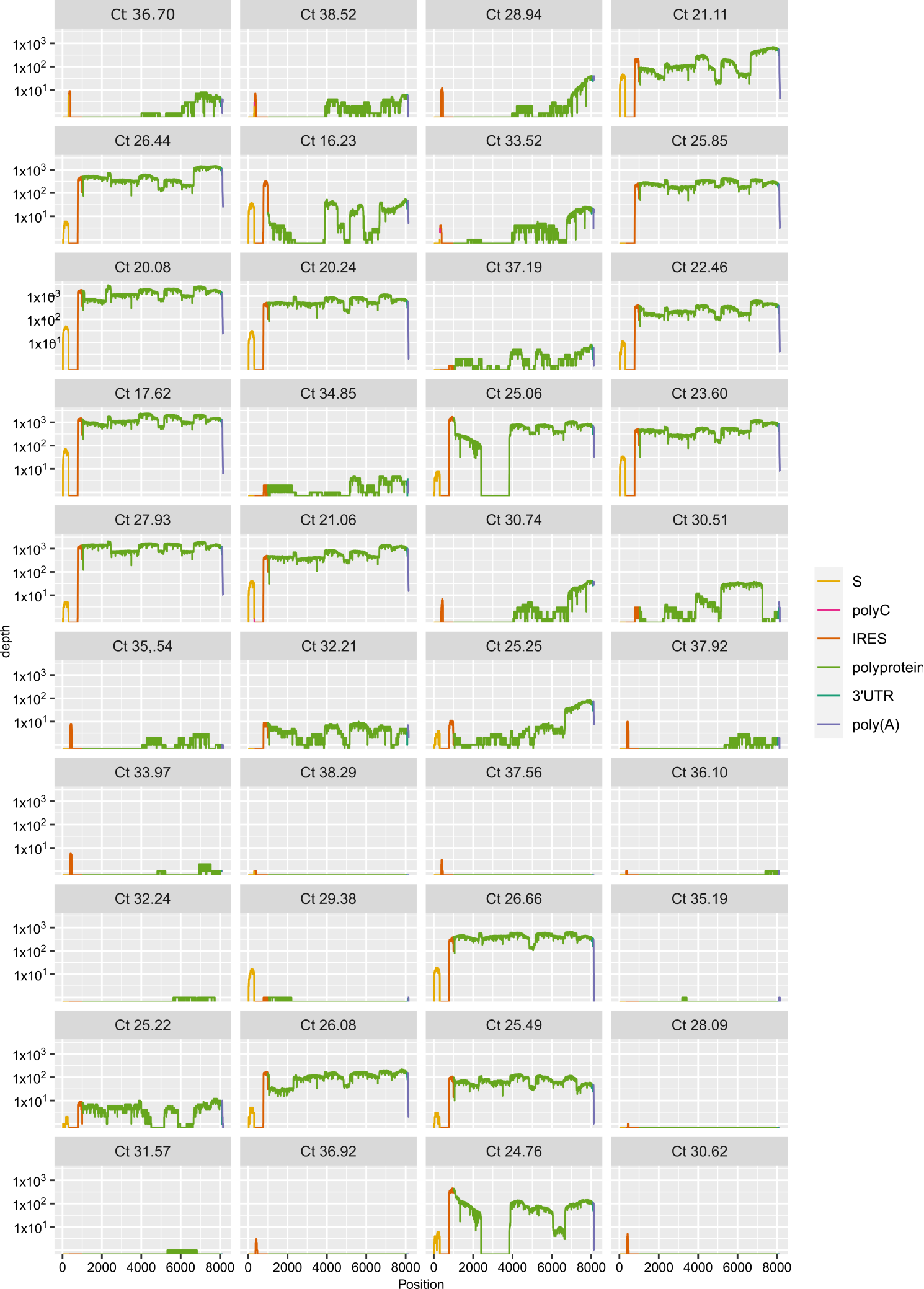

Supplement: Supplementary file 4 — Supplementary Material 4. [file 12864_2025_11938_MOESM4_ESM.pdf]

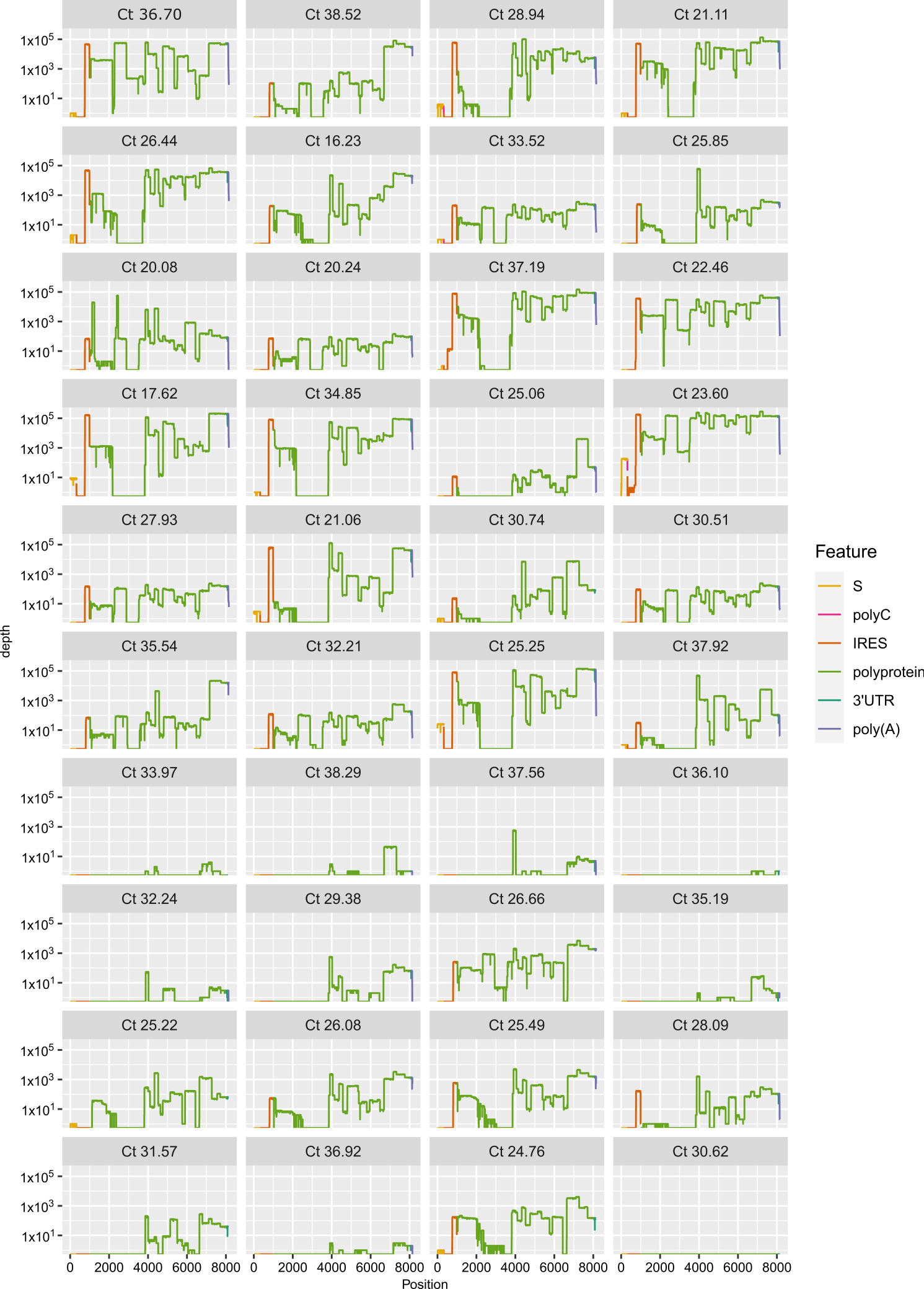

Supplement: Supplementary file 5 — Supplementary Material 5. [file 12864_2025_11938_MOESM5_ESM.pdf]

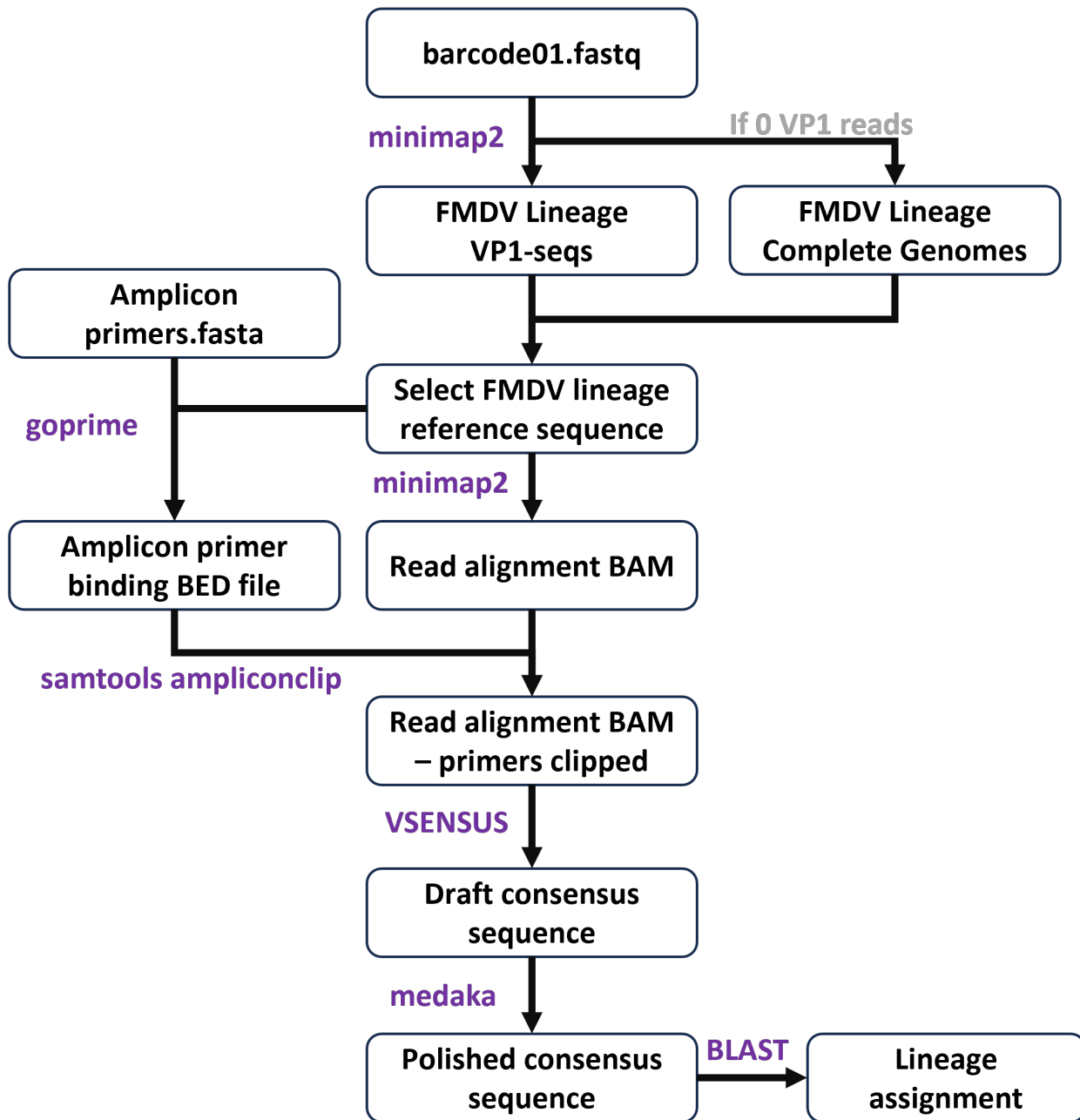

Supplement: Supplementary file 12 — Supplementary Material 12. [file 12864_2025_11938_MOESM12_ESM.pdf]

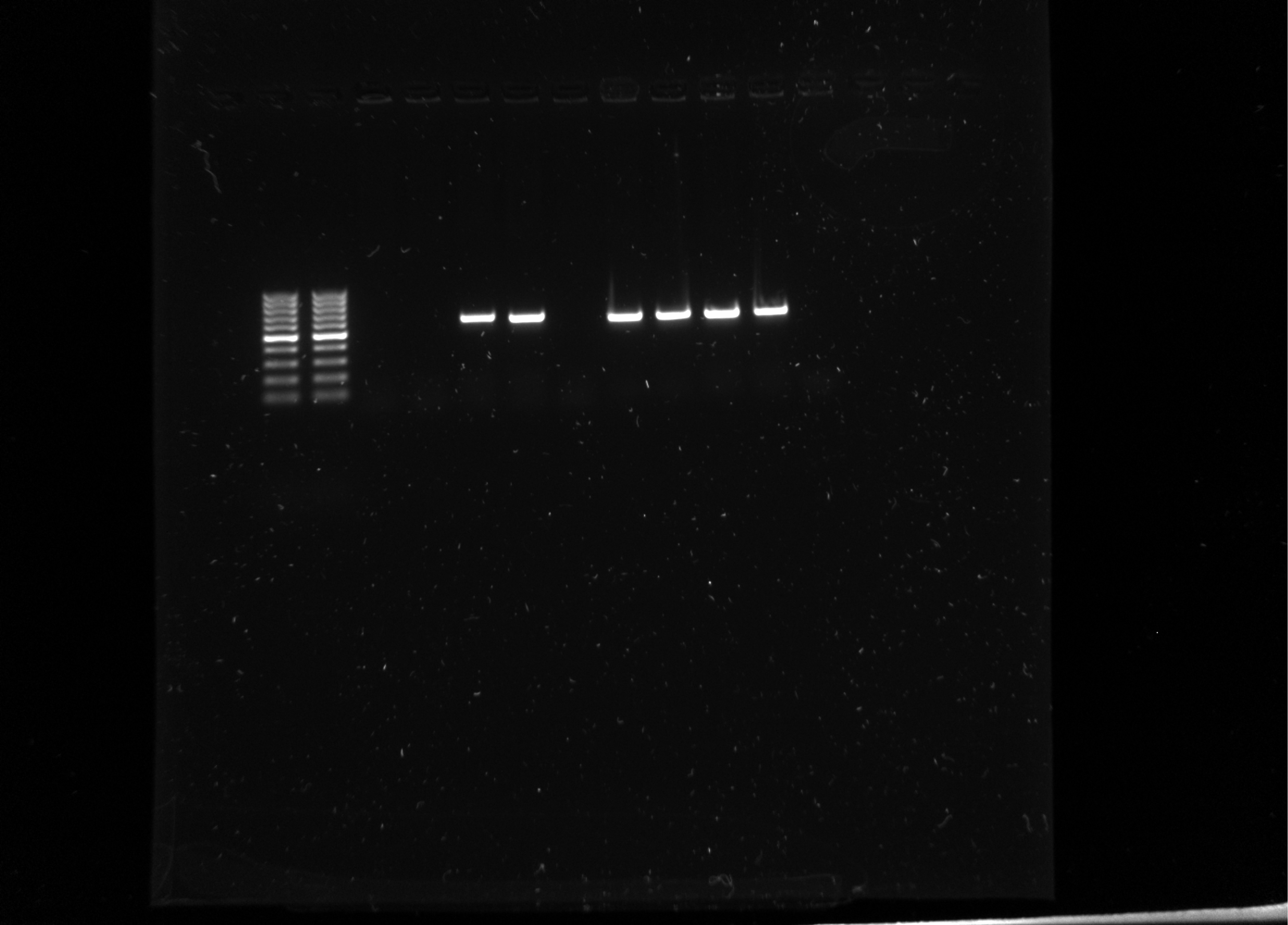

Supplement: Supplementary file 13 — Supplementary Material 13. [file 12864_2025_11938_MOESM13_ESM.png]

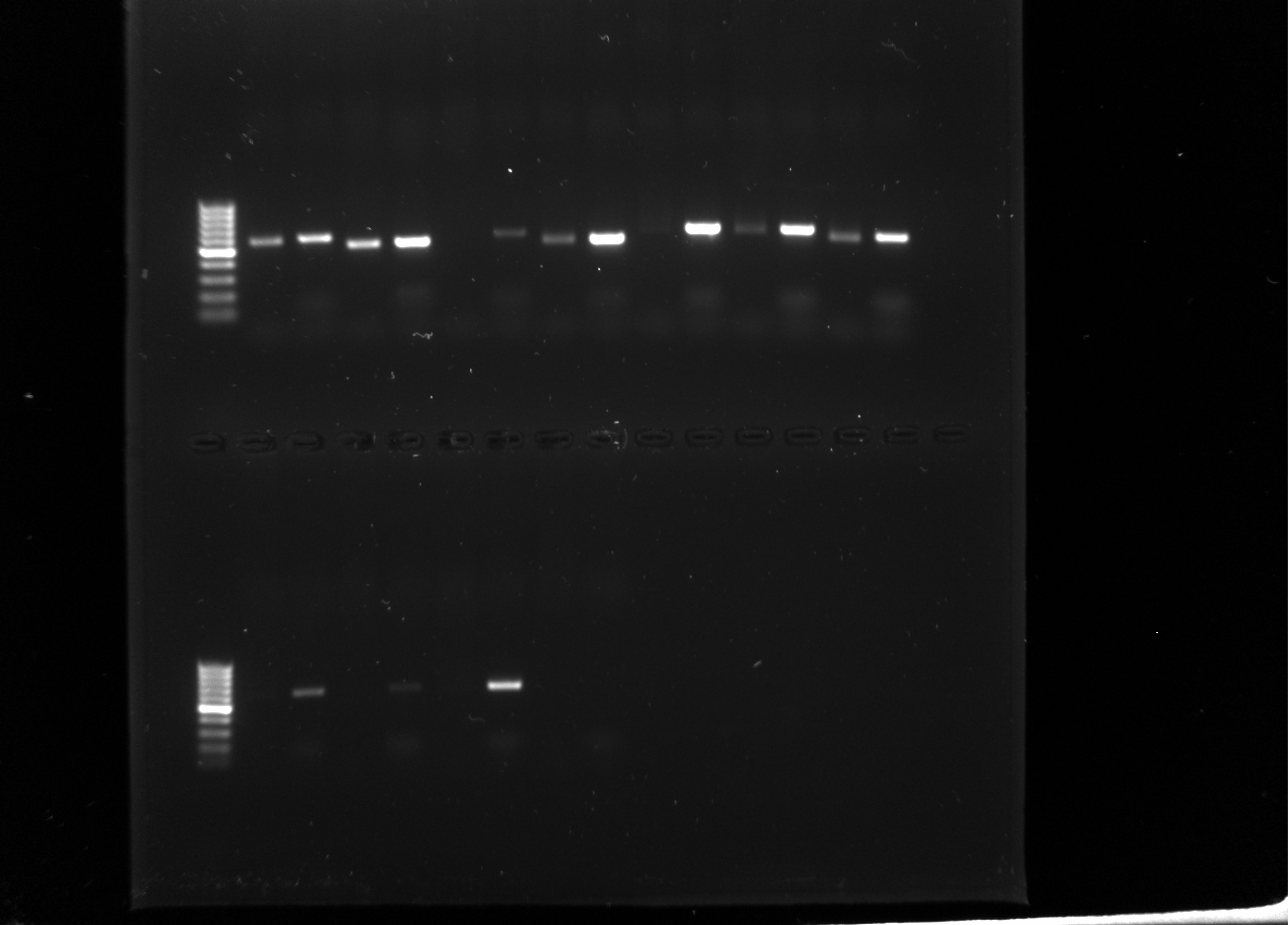

Supplement: Supplementary file 14 — Supplementary Material 14. [file 12864_2025_11938_MOESM14_ESM.png]

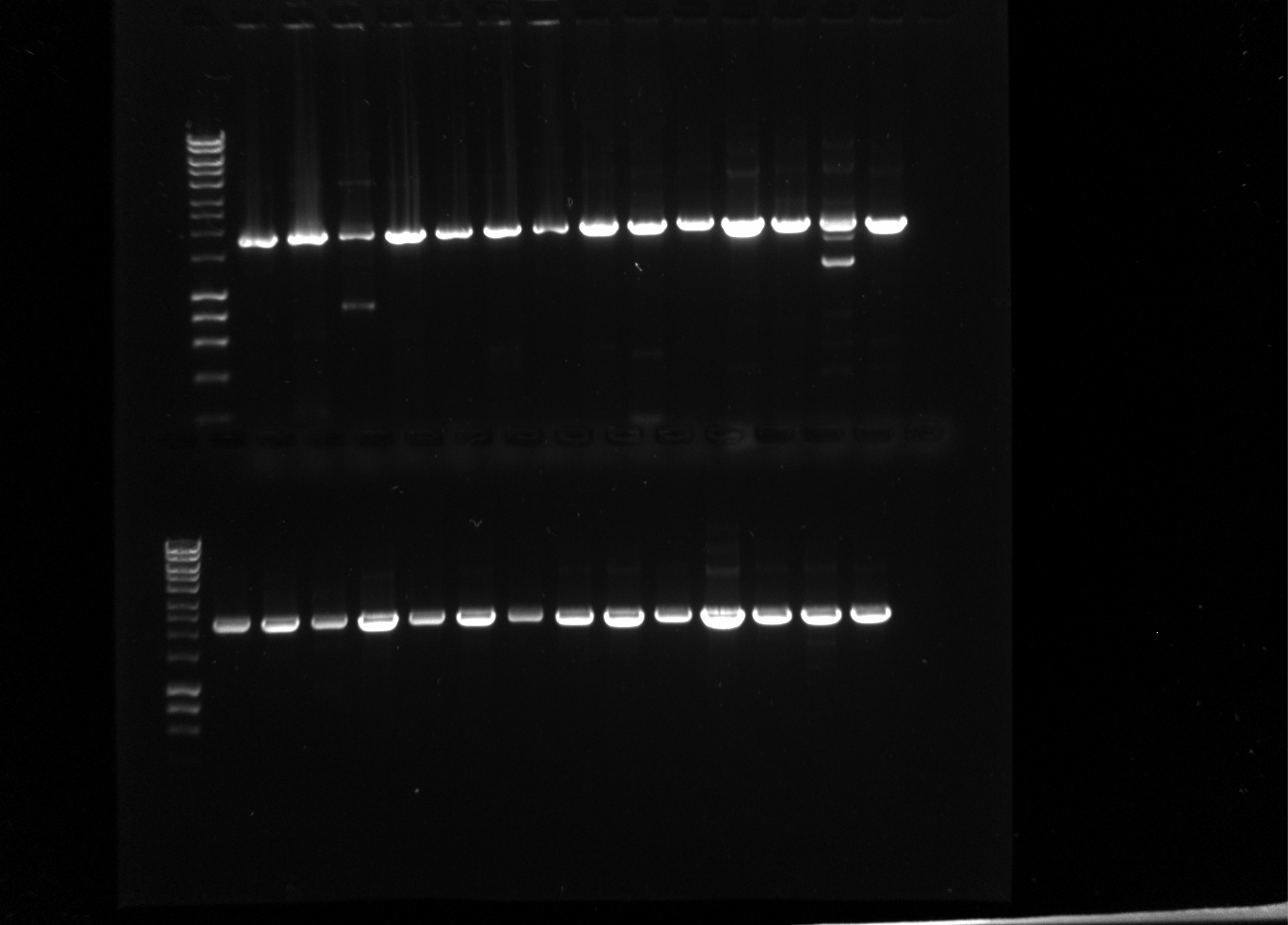

Supplement: Supplementary file 15 — Supplementary Material 15 [file 12864_2025_11938_MOESM15_ESM.png]

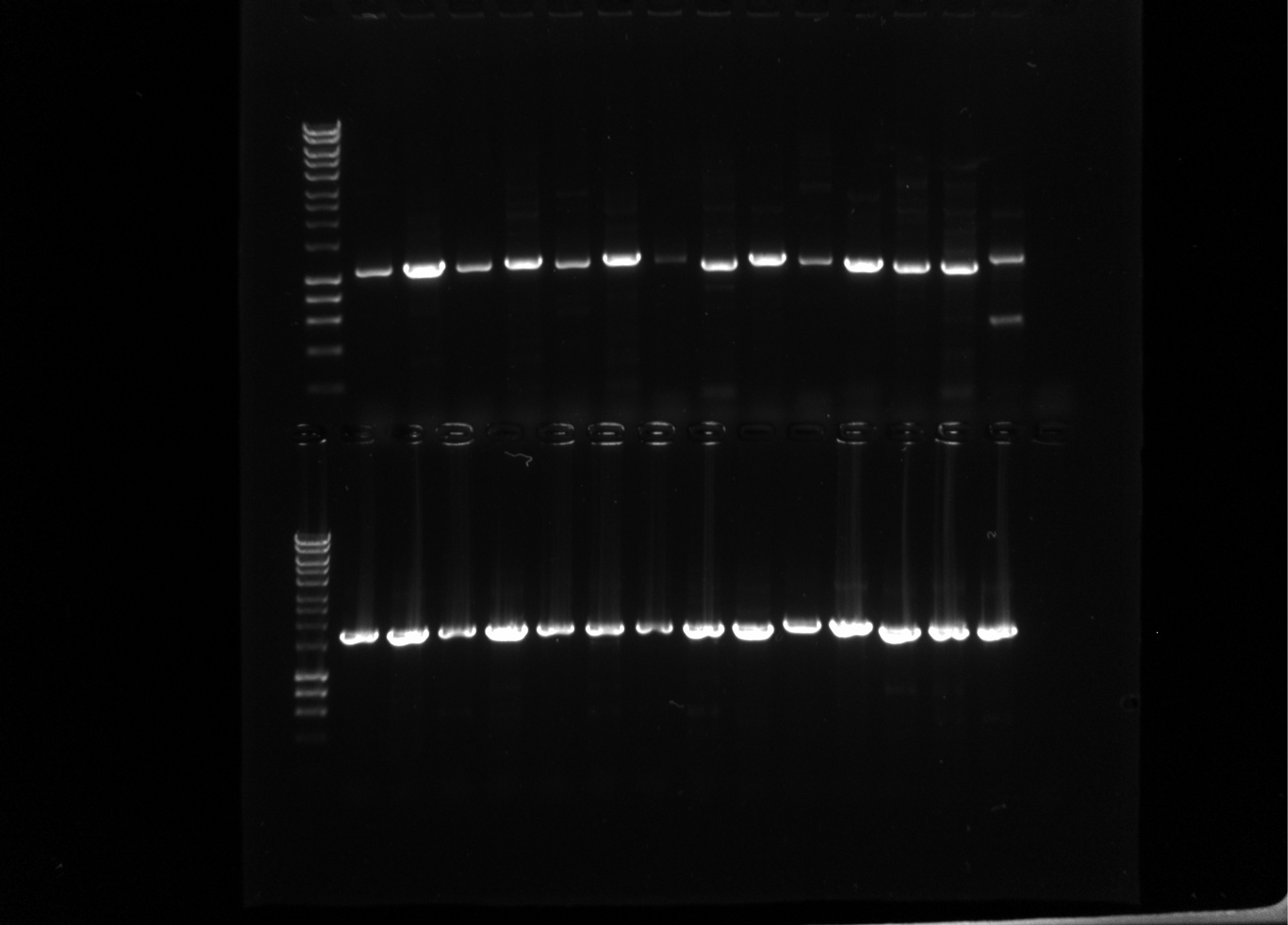

Supplement: Supplementary file 16 — Supplementary Material 16. [file 12864_2025_11938_MOESM16_ESM.png]

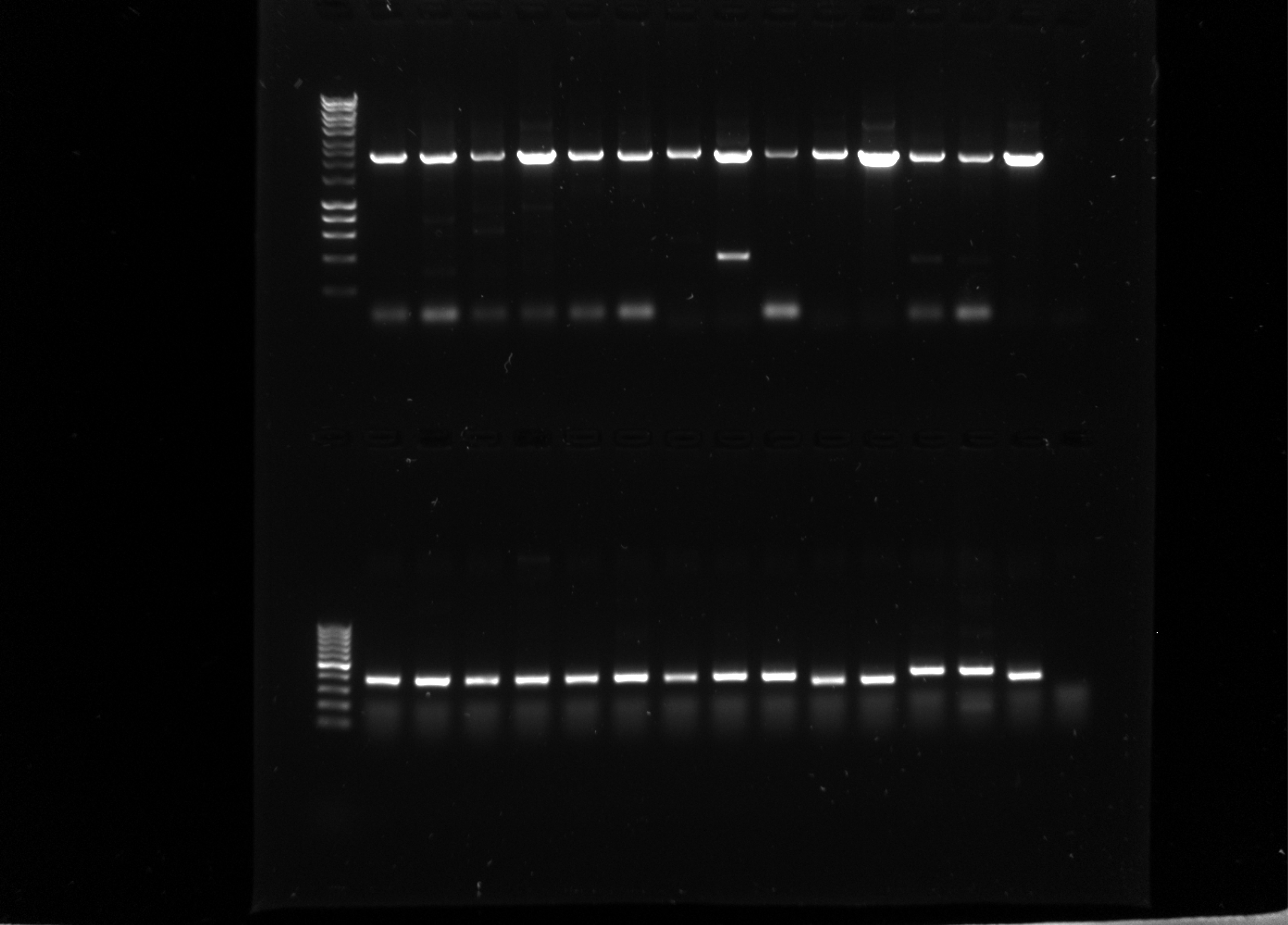

Supplement: Supplementary file 17 — Supplementary Material 17. [file 12864_2025_11938_MOESM17_ESM.png]
