# Supplementary material for: Universal amplification and sequencing of foot-and-mouth disease virus complete genomes using nanopore technology
Source: BMC Genomics. 2025 Aug 22;26:770. doi: 10.1186/s12864-025-11938-7 (PMC12372193; doi:10.1186/s12864-025-11938-7)
Supplement: Supplementary file 6 — Supplementary Material 6. [file 12864_2025_11938_MOESM6_ESM.pdf]

Table S1. PCR outcome for initial S_scheme primer designs for each amplicon targeting a panel of East African (pool 4) FMDV isolates.

Virus isolate	Lineage	S	1	2	3	**4***	**5**	**6**	**7**	**8**	**9**	10	11	12	13	14	15	16	17	18	19
KEN/4/2018	O/EA-2	+	+	+	+	+	+	+	+	+	+	+	+	+	+	+	+	+	+	+	+
ETH/4/2015	O/EA-3		+	+	+	+	+	+	+	+	+	+	+	+	+	+	+		+	+	+
ETH/9/2019	O/EA-3	+	+	+		+	+	+	+	+	+	+	+	+	+	+	+	+	+	+	+
ETH/30/2016	O/EA-4		+	+	+	+	+	+	+	+	+	+	+	+	+	+	+	+	+	+	+
ETH/14/2019	O/EA-4	+	+	+	+	+	+	+	+	+	+	+	+	+	+	+	+	+	+	+	+
ETH/2/2018	A/G-I	+	+	+	+	+	+	+	+	+	+	+	+	+	+	+	+	+	+	+	+
UGA/28/2019	A/G-I	+	+	+	+	+	+	+	+	+/-	+	+	+	+	+	+	+	+	+	+	+
SUD/9/2018	A/G-IV	+	+	+	+	+	+	+	+	+/-	+	+	+	+	+	+	+	+	+	+	+
ETH/19/2019	A/G-IV	+	+/-	+	+	+	+	+	+	+	+	+	+	+	+	+	+	+	+	+	+
TAN/27/2012	SAT1/I	+	+	+	+			+			+/-	+	+	+	+	+	+	+	+	+	+
TAN/22/2013	SAT1/I	+	+/-	+	+		+/-	+			+	+	+	+	+	+	+	+	+	+	+
KEN/10/2013	SAT1/I	+	+	+	+		+/-	+			+	+	+	+	+	+	+	+	+	+	+
TAN/22/2014	SAT1/I	+	+	+	+		+	+			+	+	+	+	+	+	+	+	+	+	+
KEN/19/2017	SAT2/IV	+	+/-	+	+	+		+	+	+	+	+	+	+	+	+	+	+	+	+	+
ETH/16/2015	SAT2/VII-Alx12	+	+	+	+	+	+	+	+	+	+	+	+	+	+	+	+	+	+	+	+
EGY/1/2018	SAT2/VII-Ghb12	+	+	+	+		+	+	+	+	+	+	+	+	+	+	+	+	+	+	+
ETH/11/2018	SAT2/VII-Lib12	+	+	+	+	+	+	+	+	+	+	+	+	+	+	+	+	+	+	+	+

*Bold typeface indicates amplicons for which at least one primer is located within the outer capsid coding genes (1B,1C and 1D).
