# Supplementary material for: Universal amplification and sequencing of foot-and-mouth disease virus complete genomes using nanopore technology
Source: BMC Genomics. 2025 Aug 22;26:770. doi: 10.1186/s12864-025-11938-7 (PMC12372193; doi:10.1186/s12864-025-11938-7)
Supplement: Supplementary file 7 — Supplementary Material 7. [file 12864_2025_11938_MOESM7_ESM.pdf]

```
library(ape)
library(dplyr)
library(tidyr)
library(stringr)

#read in alignment as a matrix
alignment<-read.dna("XXXX.fasta",format="fasta",as.character =
TRUE,as.matrix = TRUE)

#specify primer details, e.g. a primer ATGCAYGC located at position
4343-4349 of the alignment.

primer_name<-'FMDV_For'
  primer_seq<-'ATGCAYGC'
  from<-4342
  to<-4349
  refSeq<-alignment[1:1,paste(from):paste(to)]
  dput(as.character(refSeq))

  refSeq  <-  c("a", "t", "g", "c","a", "t", "g", "c")
  refSeq2 <-  c("a", "t", "g", "c","a", "c", "g", "c")
  #refSeq3 <-  c()
  #refSeq4 <-  c()

############################
#run one of the following sections according to level of redundancy.

#for no redundancies

matSeq<-alignment[1:nrow(alignment),paste(from):paste(to)]
outSeq <- matSeq
for (i in 1:ncol(matSeq)) {
  outSeq[which(matSeq[, i]==refSeq[i]), i] <- "."
}

region<-as.data.frame(outSeq)
primer<-unite(region,primer,V1:ncol(region),sep="",remove=TRUE,na.rm
= FALSE)
primer_tallys<-table(unlist(primer))
write.table(primer_tallys,"primer_tallys.csv",sep=" ")
df<-read.csv("primer_tallys.csv",sep=" ")
df<-dplyr::arrange(df, desc(Freq))
colnames(df)[1]<-paste(primer_seq)
colnames(df)[2]<-paste(primer_name)
head(df,50)


#for 2-fold redundancies e.g. R, Y, M


matSeq<-alignment[1:nrow(alignment),paste(from):paste(to)]
```

```
outSeq <- matSeq
for (i in 1:ncol(matSeq)) {
  outSeq[which(matSeq[, i]==refSeq[i]|
               matSeq[, i]==refSeq2[i]), i] <- "."
}

region<-as.data.frame(outSeq)
primer<-unite(region,primer,V1:ncol(region),sep="",remove=TRUE,na.rm
= FALSE)
primer_tallys<-table(unlist(primer))
write.table(primer_tallys,"primer_tallys.csv",sep=" ")
df<-read.csv("primer_tallys.csv",sep=" ")
df<-dplyr::arrange(df, desc(Freq))
colnames(df)[1]<-paste(primer_seq)
colnames(df)[2]<-paste(primer_name)
head(df,50)


#for 3-fold redundancies e.g. V, H


matSeq<-alignment[1:nrow(alignment),paste(from):paste(to)]
outSeq <- matSeq
for (i in 1:ncol(matSeq)) {
  outSeq[which(matSeq[, i]==refSeq[i]|
               matSeq[, i]==refSeq2[i]|
               matSeq[, i]==refSeq3[i]), i] <- "."
}

region<-as.data.frame(outSeq)
primer<-unite(region,primer,V1:ncol(region),sep="",remove=TRUE,na.rm
= FALSE)
primer_tallys<-table(unlist(primer))
write.table(primer_tallys,"primer_tallys.csv",sep=" ")
df<-read.csv("primer_tallys.csv",sep=" ")
df<-dplyr::arrange(df, desc(Freq))
colnames(df)[1]<-paste(primer_seq)
colnames(df)[2]<-paste(primer_name)
head(df,50)


#for N

matSeq<-alignment[1:nrow(alignment),paste(from):paste(to)]
outSeq <- matSeq
for (i in 1:ncol(matSeq)) {
  outSeq[which(matSeq[, i]==refSeq[i]|
               matSeq[, i]==refSeq2[i]|
               matSeq[, i]==refSeq3[i]|
               matSeq[, i]==refSeq4[i]), i] <- "."
}
```

```
region<-as.data.frame(outSeq)
primer<-unite(region,primer,V1:ncol(region),sep="",remove=TRUE,na.rm
= FALSE)
primer_tallys<-table(unlist(primer))
write.table(primer_tallys,"primer_tallys.csv",sep=" ")
df<-read.csv("primer_tallys.csv",sep=" ")
df<-dplyr::arrange(df, desc(Freq))
colnames(df)[1]<-paste(primer_seq)
colnames(df)[2]<-paste(primer_name)
head(df,50)
```
