# Supplementary material for: Universal amplification and sequencing of foot-and-mouth disease virus complete genomes using nanopore technology
Source: BMC Genomics. 2025 Aug 22;26:770. doi: 10.1186/s12864-025-11938-7 (PMC12372193; doi:10.1186/s12864-025-11938-7)
Supplement: Supplementary file 8 — Supplementary Material 8. [file 12864_2025_11938_MOESM8_ESM.pdf]

Amplicon 1 (reverse primer, pool A)

GCYTCRCGRCCCAGYGAGGTGT	UNI_Rev_2.3
....	1046
.................a....	186
...........t..........	86
........c.............	41
....................a.	29
........t.............	27
........c..t..........	15
...........t.....a....	12
........c..t.....a....	12
........t..t..........	7
....................t.	6
........c........a....	5
ag.a.t..c.........a.c.	82
ag.a.t..c.........a.t.	13
ag.a.t..c..t......a.c.	13
ag.a.t..c..a......a.c.	8

ACTCGCCCYAGCGAGATCTG	UNI_Rev_2.4
....	98
.................t..	17
........a...........	8
t.g..a.........g.g..	685
t.g..a........ag.g..	156
t.a..a.....t...g.g..	153
t.g..g.........g.g..	142
t.g..a.....t...g.g..	116
t.a........t...g.g..	36
t.a..t.....t...g.g..	30
t.g..a.........g.a..	24
t.a..g.....t...g.g..	22
t.g..g........ag.g..	17
t.g..g.....t...g.g..	17
t.g...........ag.g..	16
t.a..a.....t..ag.g..	14
t.g............g.g..	12
t.g..g.........g.a..	6
t.a........t...g.a..	5
t.g..a.........g.t..	5

Amplicon 3 (forward primer, pool A, serotype O viruses)

CGGACGAACATGACRGCVCACAT	O_UNI_For_6
....	525
..c....................	114
..c...........t........	97
.....a.................	38
..a....................	21
....................t..	10
.................t.....	10
........t..............	8
..c..a........t........	5

Amplicon 3 (forward primer, pool A, serotype A viruses)

AGAACMAACATGACWGCVCACAT	A_UNI_For_6.1
....	176
..g....................	108
.....t.................	95
.................t.....	24
..............c........	19
..g...........g........	7
..............c..t.....	6

Amplicon 3 (forward primer, pool A, serotype Asia1 viruses)

CACGCACYAACATGACGGCYCACAT	As1_UNI_For_6
....	56
.g.......................	12
....t..............a.....	7
....t..............g.....	7

Amplicon 3 (forward primer, pool A, recent serotype SAT1 viruses)

ATRAACCCGCGYACCAACACCA	SAT1_UNI_For_6A
....	9

CAATTCCTCAAYCCRCGRACGAACAC	SAT1_UNI_For_6B
....	6

Amplicon 3 (forward primer, pool A, serotype SAT2 viruses)

TACCCRCACCAGTTCATYAACCC	SAT2_UNI_For_6.1
....	74
...........a...........	6
........t..............	6
..t....................	6
....................t..	5
.....c.................	5

Amplicon 3 (forward primer, pool A, serotype SAT3 viruses)

TACCCRCACCAGTTCATYAACCC	SAT2_UNI_For_6.1
.t...t.................	13
.t.....................	7

Amplicon 3 (reverse primer, pool A)

TGTCRTGYATGGCCGCTGTRGC	UNI_Rev_9
....	1344
...................c..	141
......c...............	100
...............t......	37
...................t..	10
...........a..........	8
.............t........	6
....t........t........	5

Amplicon 5 (forward primer, pool A)

GAAGAARCCTGTCGCYTTGAARGTGA	UNI_For_15.1
....	1290
............t.............	123
...a......................	50
c.........................	42
..g.......................	29
...........c..............	28
a.........................	26
................c.........	9
.....g....................	6
........................a.	5
.........c................	5
c..........c..............	5

Amplicon 2 (forward primer, pool B)

CRTGTGTGCRACCCCRGCAC	UNI_For_2
....	1208
t...................	192
...........a........	15
...........t........	10
t...c...............	7
..c.................	6
t.c.................	6
..............t.....	5
..........g.........	5
t.............t.....	5

Amplicon 2 (reverse primer, pool B, serotype O viruses)

ATYAAGGTBTAYGCCAACATCGCCCC	O_A_UNI_Rev_5
....	393
....................t.....	166
.......................a..	165
.................t........	31
..............t...........	23
........a.................	12
.......................g..	10
.......................t..	10
....................a.....	8
..............t........g..	6
..............t.....t.....	6
........a.....t...........	5

Amplicon 2 (reverse primer, pool B, serotype A viruses)

ATYAAGGTBTAYGCCAACATCGCCCC	O_A_UNI_Rev_5
....	180
....................a..t..	88
....................t.....	61
.......................t..	42
....................t..t..	12
..............t...........	11
.....a....................	10
.....a..............t.....	9
.................t........	7

Amplicon 2 (reverse primer, pool B, serotype Asia1 viruses)

ATYAAGGTYTACATGAATGCAGCACC	As1_UNI_Rev_5.2
....	41
.......................g..	32
....................t.....	10

Amplicon 2 (reverse primer, pool B, recent serotype SAT1 viruses)

TTCCAAAACACDGACCCCAARAC	SAT1_UNI_Rev_5A
....	16
..........c....g.......	5

CCAAAACACCGACCCGAAGACTT	SAT1_UNI_Rev_5A.1
....	5
........a.....c..a..a..	7

Amplicon 2 (reverse primer, pool B, serotype SAT2 viruses)

GTYGAGGTGTACGCCAACATYGC	SAT2_UNI_Rev_5
....	47
..............t........	17
........c..............	11
........t..............	9
........a..............	5

Amplicon 2 (reverse primer, pool B, serotype SAT3 viruses)

TTCCAAAACACDGACCCCAARAC	SAT1_UNI_Rev_5A
.....g.................	11
.....g...........a.....	8
..............t........	6

GTYGAGGTGTACGCCAACATYGC	SAT2_UNI_Rev_5
..............a........	10
........c..............	5
..a........t..g........	5

Amplicon 4 (forward primer, pool B)

CCAACCCTGGRCCCTTCTTYTT	UNI_For_10
....	1308
.......c..............	105
.............t........	94
....t.................	55
.......a..............	29
................t.....	16
.t....................	11
....t........t........	6

Amplicon 4 (reverse primer, pool B)

GGBAAGACRGTRGCCATCTGCTG	UNI_Rev_14A
....	905
.................t.....	250
..............a........	116
....................t..	95
..............g........	28
..............t........	23
.................t..t..	10
...............g.......	9
........c..t..gc.......	48
...........t..gc.......	38
...........t..ac.t.....	9
........c..t..ac.g.....	9
.......tc..c..gc.......	9
...............g....t..	5
...........t..gc.g.....	5

GGKAAGACVGTTGCRCTCTGCTG	UNI_Rev_14B
....	89
.................g.....	16
.................t.....	12
....................t..	6
...........c...........	6
...........a..ca.......	560
...........g..ca.......	317
...........a..ca.t.....	203
...........g...a.......	127
...........a..ca....t..	46
...........g..ca.t.....	35
...........g..ca....t..	34
..c........a..ca.......	23
...........g..ta.......	18
...........a...a.......	15
..c........g..ca....t..	15
.......t...c...........	9
...........g..ca.t..t..	8
..c........a..ca.t.....	8
...........g..cg.......	7
...........g...a....t..	6
...........a..ta.......	5
...........g..cg....t..	5
..c........g..ca.......	5

GGKAAGACMGTKGCRMTCTGCTG	UNI_Rev_14C
....	208
..............c........	70
..............t........	19
.................g.....	16
...........a...........	15
........g..............	13
....................t..	12
........g........t.....	9
..............c.....t..	6
..............c..t.....	6
........t..............	6
.................t.....	5
...........a..c........	461
........g.....c........	251
...........a..c..t.....	109
........g..a..c........	99
........g..a..c..t.....	94
........g.....c..t.....	29
........g.....c.....t..	28
...........a..c.....t..	26
........g..a..c.....t..	20
..c.....g.....c.....t..	15
..c.....g..a..c........	13
..c........a..c........	10
.......t...c...........	9
..c.....g..a..c..t.....	8
........g.....cg.......	7
........g.....c..t..t..	6
..............cg....t..	5
