# Supplementary material for: Universal amplification and sequencing of foot-and-mouth disease virus complete genomes using nanopore technology
Source: BMC Genomics. 2025 Aug 22;26:770. doi: 10.1186/s12864-025-11938-7 (PMC12372193; doi:10.1186/s12864-025-11938-7)
Supplement: Supplementary file 9 — Supplementary Material 9. [file 12864_2025_11938_MOESM9_ESM.pdf]

Table S3. Primer counts per S_scheme amplicon mix.

Amplicon_mix	No. primers
S	2
1	7
2	3
3	3
4	10
5	10
6	11
7	13
8	11
9	9
10	2
11	2
12	2
13	3
14	7
15	3
16	5
17	4
18	2
19	2
