# Supplementary material for: Universal amplification and sequencing of foot-and-mouth disease virus complete genomes using nanopore technology
Source: BMC Genomics. 2025 Aug 22;26:770. doi: 10.1186/s12864-025-11938-7 (PMC12372193; doi:10.1186/s12864-025-11938-7)
Supplement: Supplementary file 10 — Supplementary Material 10. [file 12864_2025_11938_MOESM10_ESM.pdf]

Virus	Ct
A/IRN/24/2015	25.70
A/KEN/10/2021	25.40
A/NEP/5/2021	21.90
A/NIG/87/2020	22.20
A/PAK/1/2020	20.60
A/PAK/30/2021	29.80
A/VIT/19/2017	19.90
Asia1/PAK/47/2021	17.30
O/CAM/3/2018	21.00
O/COD/43/2021	20.80
O/GHA/1/2016	22.00
O/HKN/1/2019	21.80
O/ISR/5/2021	14.00
O/JOR/2/2017	21.60
O/PAT/8/2021	16.70
O/SRL/13/2019	23.40
O/UAE/2/2021	14.50
O/VIT/47/2018	19.90
SAT1/KEN/3/2020	23.20
SAT1/QTR/4/2023	12.27
SAT1/TAN/27/2012	19.36
SAT2/EGY/17/2012	26.10
SAT2/NIG/5/2021	16.90
SAT2/NIG/57/2020	19.80
SAT2/NIG/91/2020	17.00
SAT2/SUD/12/2017	19.00
SAT3/ZAM/9/2018	15.00
