# Supplementary material for: Universal amplification and sequencing of foot-and-mouth disease virus complete genomes using nanopore technology
Source: BMC Genomics. 2025 Aug 22;26:770. doi: 10.1186/s12864-025-11938-7 (PMC12372193; doi:10.1186/s12864-025-11938-7)
Supplement: Supplementary file 11 — Supplementary Material 11. [file 12864_2025_11938_MOESM11_ESM.pdf]

Amplicon 1 (reverse primer)

GCYTCRCGRCCCAGYGAGGTGT	UN_Rev_2.3
....	32
.....................g	2
....................a.	2
........c.............	2
........t...........a.	1

Amplicon 2 (forward primer)

CRTGTGTGCRACCCCRGCAC	UNI_For_2
....	40
....c.....tt........	2

Amplicon 2 (reverse primer)

ATYAAGGTBTAYGCCAACATCGCCCC	O_A_UNI_Rev_5
.....a..............a.....	26
....................a.....	5
..............a.....a.....	3
g....a..............a.....	3
g...................a.....	2
.................t..a.....	1

Amplicon 3 (forward primer)

CGGACGAACATGACRGCVCACAT	O_UNI_For_6
..c..c.................	24
..t..t.................	6
..c..t.................	3
..c..c..t..............	2
..t..a.................	2
..a..t.................	1
..c..a.................	1
..t..c.................	1

Amplicon 3 (reverse primer)

TGTCRTGYATGGCCGCTGTRGC	UNI_Rev_9
....	41
..............a.......	1

Amplicon 4 (forward primer)

CCAACCCTGGRCCCTTCTTYTT	UNI_For_10
....	37
.......c..............	2
....t.................	2
.............a........	1

Amplicon 4 (reverse primer)

GGBAAGACRGTRGCCATCTGCTG	UNI_Rev_14A
....	37
....................t..	3
..............a........	1
..a.................t..	1

Amplicon 5 (forward primer)

GAAGAARCCTGTCGCYTTGAARGTGA	UNI_For_15.1
....	39
................c.........	2
...a......................	1
