# Supplementary material for: Universal amplification and sequencing of foot-and-mouth disease virus complete genomes using nanopore technology
Source: BMC Genomics. 2025 Aug 22;26:770. doi: 10.1186/s12864-025-11938-7 (PMC12372193; doi:10.1186/s12864-025-11938-7)
Supplement: Supplementary file 18 — Supplementary Material 18. [file 12864_2025_11938_MOESM18_ESM.docx]

**Additional file legends**

**Additional file 1.**Table S1. PCR outcome for initial S_scheme primer designs for each amplicon targeting a panel of East African (pool 4) FMDV isolates.

**Additional file 2.** Table S2. PCR outcome for initial S_scheme primer designs for each amplicon targeting a panel of Southeast Asian (pool 1) FMDV isolates.

**Additional file 3.** Representative coverage plots for clinical swab samples collected in Uganda sequenced using the initial L_scheme arrangement. Lines are coloured according to the region of the genome. Real-time RT-PCR Ct values are those of the pan-FMDV 3D Callahan assay.

**Additional file 4.** Representative coverage plots for clinical swab samples collected in Uganda sequenced using the initial S_scheme arrangement. Lines are coloured according to the region of the genome. Real-time RT-PCR Ct values are those of the pan-FMDV 3D Callahan assay.

**Additional file 5.** Coverage plots for environmental swab samples collected in Uganda from a herder’s stick (left) or a tethering rope (right). Samples were sequenced using the original L_scheme (upper) or S_scheme (lower) strategies. Lines are coloured according to the region of the genome.

**Additional file 6.** The complete VP1 tree shown as a cropped version in Figure 6.

**Additional file 7.** The R script used to subset an alignment (matrix) and tabulate the frequency of each target sequence.

**Additional file 8.** L_scheme primer footprint matches for sequences represented at a frequency of 5 or greater. Sequences shaded grey are encompassed by an alternative primer. All primers are in genome sense, i.e. reverse primers are not reverse complemented. Primers for amplicons 6 and 5 used alignments of individual serotypes. Pool A and Pool B refer to the primer pools assembled for the final L_scheme.

**Additional file 9.** Table S3. Primer counts per S_scheme amplicon mix.

**Additional file 10.** L_scheme primer conservation relative to serotype C genomes. All primers are in genome sense (i.e. reverse primers are not reverse complemented). 42 complete genomes were available.

**Additional file 11.** Real-time PCR FMDV Ct values for the cDNA samples used to evaluate the final sequencing protocol. Values were generated using an assay targeting the 3D region of the FMDV genome [56].

**Additional file 12.** Schematic diagram of the NanoFMDV pipeline to assemble consensus sequences from fastq data in the absence of a priori information regarding serotype/lineage.
